# Deletion of *TaBSK3‐2B* attenuates BR signaling to regulate the *Rht4* semi‐dwarf phenotype and drought tolerance in wheat

**DOI:** 10.1111/tpj.71118

**Published:** 2026-09-17

**Authors:** Qiumei Lu, Shan Lu, Zining Wang, Yihan Men, Renmei Tian, Yue Qiao, Xianglan Kong, Zhangchen Zhao, Chunge Cui, Yanju Huang, Liang Chen, Yin‐gang Hu, Linzhou Huang

**Affiliations:** ^1^ State Key Laboratory of Crop Stress Resistance and High Efficiency Production, College of Agronomy Northwest A&F University Yangling Shaanxi 712100 China; ^2^ Institute of Water Saving Agriculture in Arid Regions of China Northwest A&F University Yangling Shaanxi 712100 China

**Keywords:** *Rht4*, semi‐dwarfing gene, plant height, BR signaling, drought tolerance, yield

## Abstract

The application of Green Revolution alleles *Rht‐B1b* and *Rht‐D1b* in wheat semi‐dwarf breeding has remarkably improved lodging resistance and increased global wheat production. However, these gibberellin (GA)‐insensitive genes require higher water and fertilizer inputs and impair seedling emergence and early vigor, prompting the search for alternative GA‐sensitive dwarfing genes to breed environmentally friendly wheat varieties for sustainable wheat production. Here, we reported the map‐based cloning of *Rht4* and functional characterization of its potential causal gene *TaBSK3‐2B*, which encodes a plasma membrane‐localized serine/threonine‐protein kinase involved in the brassinosteroid (BR) signaling pathway. Loss function of *TaBSK3‐2B* attenuated BR responses and inhibited stem elongation by reducing cell expansion, which was accompanied by decreased levels of the bioactive gibberellins GA_1_ and GA_4_. Under controlled conditions, *TaBSK3‐2B* positively regulates wheat drought tolerance by integrating transcriptional level changes of key drought‐related genes and physiological adjustments under water deficit. Furthermore, we identified a natural haplotype *TaBSK3‐2B* ^*H2*^ which was associated with reduced plant height and improved yield‐related traits, with the potential to mitigate the yield penalty associated with the original *Rht4* allele, a major limitation on its application in wheat breeding. The frequency of *TaBSK3‐2B* ^*H2*^ is dramatically higher in cultivars compared to landraces, indicating a strong artificial selection of *TaBSK3‐2B* ^*H2*^ during semi‐dwarf wheat breeding. Our study not only provides new insights into the synergistic improvement of plant height and grain yield by fine‐tuning BR signaling but also offers potential genetic resources for breeding drought‐adapted semi‐dwarf wheat varieties.

## Significance Statement

GA‐insensitive Green Revolution genes *Rht‐B1b* and *Rht‐D1b* improve lodging resistance but often demand higher inputs and compromise early vigor, limiting their performance under drought conditions. Here, we cloned *TaBSK3‐2B* as the major contributor of wheat *RHT4* locus and demonstrated that *TaBSK3‐2B* positively regulates wheat drought resistance and identified a natural allele *TaBSK3‐2B* ^*H2*^ which is associated with shorter plant height with improved yield‐related traits, providing a strategy to coordinate wheat plant height and yield by modulating BR signaling pathway.

## INTRODUCTION

Plant height is a crucial component of crop plant architecture affecting biomass accumulation, lodging resistance and yield potential (Cadalen et al., 1998; Chai et al., 2018; Miao et al., 2024). In the 1960s, the widespread adoption of the semi‐dwarfing alleles *Reduced height* (*Rht)‐B1b* (*Rht1*) and *Rht‐D1b* (*Rht2*) propelled the ‘Green Revolution’ in wheat through improving lodging resistance and dramatically increasing grain yield, thereby alleviating the global food crisis (Hedden, 2003). However, wheat cultivars carrying the classical *Rht‐B1b* or *Rht‐D1b* alleles typically require more agricultural inputs, including sufficient water supply and high nitrogen fertilizer to achieve the maximum yield potential, posing significant challenges to sustainable agriculture (Ehdaie & Waines, 1996; Gooding et al., 2012; Hedden, 2003; Jatayev et al., 2020). Additionally, these semi‐dwarf cultivars often exhibit less aboveground biomass and lower grain yield compared to taller or intermediate‐height varieties under heat or drought conditions (Jatayev et al., 2020; Uppal & Gooding, 2013; Zhao et al., 2022). Due to the reduced coleoptile length and lower early seedling vigor conferred by *Rht‐B1b* and *Rht‐D1b*, Green Revolution wheat varieties are less successful in arid and semi‐arid regions and perform even more poorly when exposed to frequent and prolonged drought (Fischer & Maurer, 1978; Monneveux et al., 2012). Consequently, identifying alternative reduced height loci that minimize adverse effects on early seedling growth has long been a major goal for breeding drought‐resilient wheat varieties.

Multiple semi‐dwarfing genes have been identified across plant species, underscoring the core role of gibberellin (GA) in controlling stem elongation and plant height (Peng et al., 1999; Robil et al., 2025; Sasaki et al., 2002). Binding of GA to its receptor GIBBERELLIN INSENSITIVE DWARF1 (GID1) triggers the recruitment of DELLA proteins to the SCF^SLY1/GID2^ complex, leading to the degradation of DELLA proteins via the ubiquitin‐26S proteasome machinery, which, in turn, eliminates the inhibitory effects of DELLAs on GA signaling (Olszewski et al., 2002; Wang et al., 2025; Yamaguchi, 2008). The N‐terminally truncated DELLA proteins encoded by the Green Revolution *Rht‐B1b* and *Rht‐D1b* are insensitive to GA‐induced degradation, thus shaping semi‐dwarfism by repressing GA signaling (van de Velde et al., 2021). Other alleles at the *RHT‐1* locus including *Rht‐B1c* (*Rht3*), *Rht‐D1c* (*Rht10*), *Rht‐B1e* (*Rht11*), and *Rht‐B1p* (*Rht17*) also confer GA‐insensitive dwarf or semi‐dwarf phenotypes through blocking GA signaling and inhibiting GA‐promoted stem elongation (Bazhenov et al., 2015; Börner et al., 1996; Divashuk et al., 2012). By contrast, several GA‐sensitive loci without adverse effects on seedling growth, such as *Rht8*, *Rht13*, and *Rht24*, have been considered as alternative dwarfing genes for breeding drought‐tolerant wheat varieties under limited water conditions and characterization of the causal genes underlying these alternative reduced height loci demonstrated their roles in maintaining GA homeostasis in wheat (Gasperini et al., 2012; Khalid et al., 2024; Tian, Xia, et al., 2022). *Rht8* was initially reported to encode a putative Ribonuclease H‐like domain protein (RNHL‐D1) that regulates stem elongation and plant height by altering the expression of GA‐related genes thus changing the levels of bioactive GAs (Chai et al., 2022; Xiong et al., 2022). Further fine mapping demonstrated that two tightly linked neighboring genes, *TraesCS2D02G057800* and *TraesCS2D02G057900*, are the causative genes underlying the *Rht8* locus (Cheng et al., 2024). In the *Rht12* background, a loss‐of‐function mutation in *TaGA2oxA13* resulted in excessive stem elongation, supporting a key role for GA catabolism in *Rht12*‐mediated dwarfism (Buss et al., 2020). Similarly, *Rht24*, which is associated with altered expression of the gibberellin 2‐oxidase gene *TaGA2oxA9*, decreases plant height without yield penalty through reducing bioactive GA levels in wheat stem (Tian, Xia, et al., 2022). A similar GA‐deactivation mechanism also underlies semi‐dwarfing phenotypes of *Rht14* and *Rht18* (Ford et al., 2018; Vikhe et al., 2019). Recent cloning of wheat dwarfing loci, including *Rht25*, *Rht13* and *Rht23*, has further illustrated the mechanistic diversity in the regulation of wheat plant height, highlighting the value of identifying alternative dwarfing genes for wheat improvement (Borrill et al., 2022; Zhang et al., 2023; Zhao et al., 2018).

Brassinosteroids (BRs) are essential regulators of plant height that regulate stem elongation primarily by promoting cell elongation, division, and differentiation. Mutants with disrupted BR biosynthesis or signaling typically exhibit pronounced dwarf or semi‐dwarf phenotypes with reduced grain size and grain yield, such as *brd1*, *d2*, *d11*, *d61*, and *dlt* (Hong et al., 2002, 2003; Tanabe et al., 2005; Tong et al., 2009; Yamamuro et al., 2000). BR signaling involves a plasma membrane‐to‐nucleus signal transduction cascade in which BR perception by the BRI1‐BAK1 receptor complex inhibits kinase BIN2, activating BES1/BZR1 transcription factors to regulate the expression of growth‐related genes (Nolan et al., 2020; Wang et al., 2012; Wang & Chory, 2006). Wheat *tabri1* mutant carrying a premature stop codon confers severe dwarfism and a compact plant architecture, highlighting the dose effect of *TaBRI1* genes in BR perception in hexaploid wheat development (Gill et al., 2025). Likewise, TaGSK3, the wheat homolog of rice BIN2, acts as a negative modulator of wheat BR signaling. It has been reported that TaGSK3 could stabilize Rht‐B1b through phosphorylation at the S92/S135/S136, thus enhancing the semi‐dwarfism through inhibiting downstream transcription factors (Dong et al., 2023). Song et al. (2023) have demonstrated that deletion of the *r*‐*e*‐*z* haploblock could stabilize the BR signaling repressor TaBKI1 to partially attenuate BR perception, therefore, conferring a compact semi‐dwarf architecture with increased grain yield. In addition, the wheat MADS‐box transcription factor PEDUNCLE LENGTH 1 (TaPL1) was also involved in BR signaling to regulate wheat peduncle elongation and yield traits (Liu et al., 2025). Collectively, these findings highlighted the potential roles of BR in coordinating wheat plant height and grain yield.

*Rht4* is a recessive dwarfing gene identified in a mutant derived from the cultivar ‘Burt’ through gamma‐ray and fast neutrons radiation (Konzak, 1976). In contrast to *Rht‐B1b* and *Rht‐D1b*, the GA‐sensitive *Rht4* does not exhibit adverse effects on coleoptile length and early seedling vigor (Ellis et al., 2004; Lu et al., 2022). Previous study has mapped *Rht4* on wheat chromosome 2BL which was closely linked to the SSR marker *Xwmc317* (Ellis et al., 2005). In this study, we isolated the wheat semi‐dwarf gene *Rht4* through map‐based cloning. We found that three genes were deleted in a ~214‐kb candidate region of the *Rht4* locus, among which *TaBSK3‐2B* encodes a serine/threonine protein kinase that localized at the plasma membrane. We showed that TaBSK3‐2B functions as a positive regulator of wheat BR signaling to regulate plant height and drought tolerance. Haplotype analysis identified a natural allele *TaBSK3‐2B* ^*H2*^ that was associated with significantly reduced plant height and improved grain yield‐related traits in wheat, providing a potentially valuable gene resource for molecular design breeding of new semi‐dwarf, high‐yield wheat varieties.

## RESULTS

### Genetic effects of *Rht4* on agronomic traits

**Figure 1:**
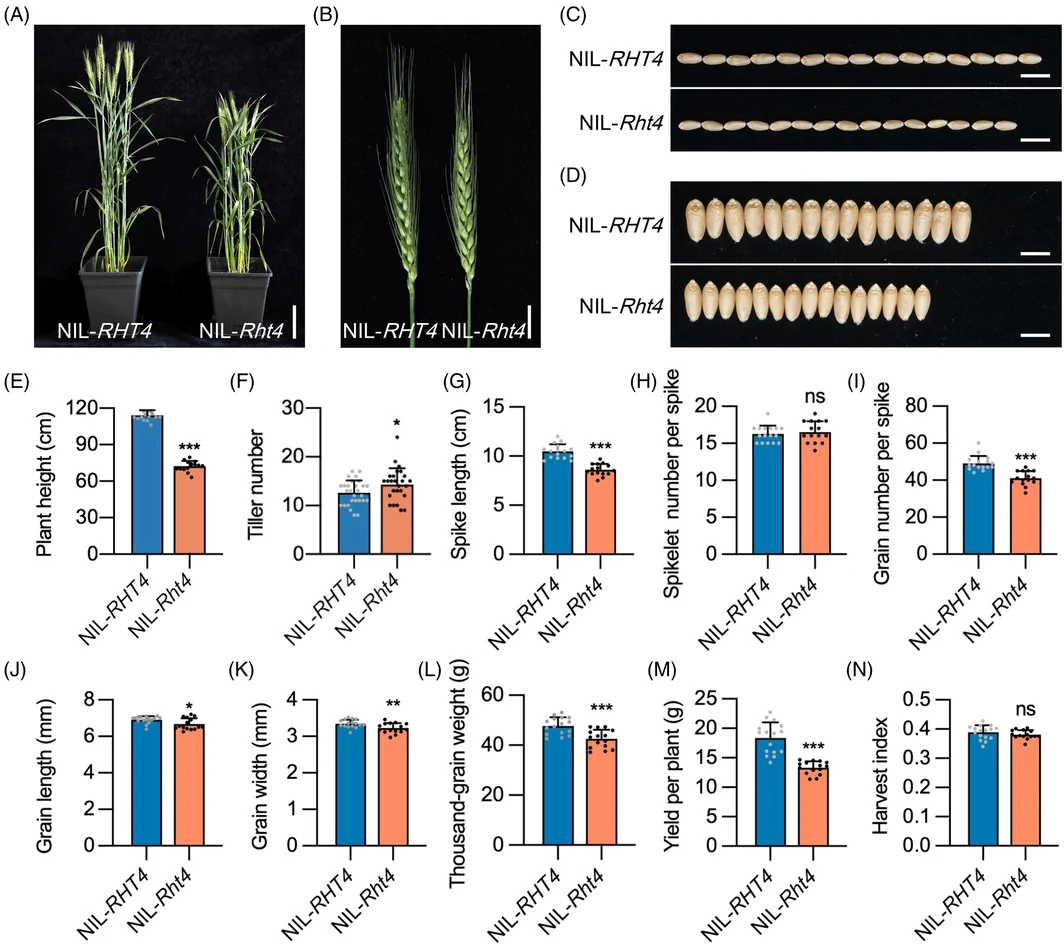
Phenotypic characterization of *Rht4* near‐isogenic lines (NILs). (A) Gross morphologies of NIL‐*RHT4* and NIL‐*Rht4* at grain‐filling stage. Bar = 10 cm. (B–D) Comparison of spike length (B), grain length (C) and grain width (D) between NIL‐*RHT4* and NIL‐*Rht4*. Bars = 2, 1 and 0.5 cm, respectively. (E–N) Statistical analysis of plant height (E), tiller number (F), spike length (G), spikelet number per spike (H), grain number per spike (I), grain length (J), grain width (K), thousand‐grain weight (L), yield per plant (M) and harvest index (N) of NIL‐*RHT4* and NIL‐*Rht4*. Data are presented as means ± SD, *n*_NIL‐*RHT4*_ = 16, *n*_NIL‐*Rht4*_ = 15 in (E, G–N), and *n*_NIL‐*RHT4*_ = 28, *n*_NIL‐*Rht4*_ = 26 in (F). *P < 0.05, **P < 0.01, ***P < 0.001, Student's *t*‐test.

To assess the phenotypic variation determined by the *Rht4* locus, near‐isogenic lines (NILs) for *Rht4* were developed from a cross between Jinmai47 (carrying the tall allele *RHT4*) and the donor Burt ert937 (carrying the semi‐dwarfing allele *Rht4*). Compared to the tall NIL‐*RHT4*, NIL‐*Rht4* plants exhibited a semi‐dwarf plant architecture, with significant reduction in plant height (Figure 1A,E), spike length (Figure 1B,G), and grain number per spike (Figure 1I) while no significant difference was observed in spikelet number (Figure 1H). Additionally, NIL‐*Rht4* plants produced shorter and smaller grains with significant reduction in grain length and width but increased roundness (Figure 1C,D,J,K, Figure S1). Although the NIL‐*Rht4* displayed more effective tillers (Figure 1F), this increase could not compensate for the remarkable decrease in grain size (Figure 1J,K) and thousand‐grain weight (Figure 1L), leading to a significantly lower grain yield per plant compared with NIL‐*RHT4* (Figure 1M). Additionally, the harvest index did not differ significantly between the two NILs (Figure 1N), implying a negative effect of *Rht4* on biomass. Importantly, no significant difference in coleoptile length was observed between NIL‐*RHT4* and NIL‐*Rht4* (Figure S2), consistent with earlier reports that *Rht4* does not compromise seedling emergence (Ellis et al., 2004; Lu et al., 2022). Together, these results demonstrated the pleiotropic role of *Rht4* in controlling wheat plant architecture and grain yield.

### *Rht4* inhibits stem elongation by modulation of gibberellin (GA)‐mediated cell proliferation and expansion

**Figure 2:**
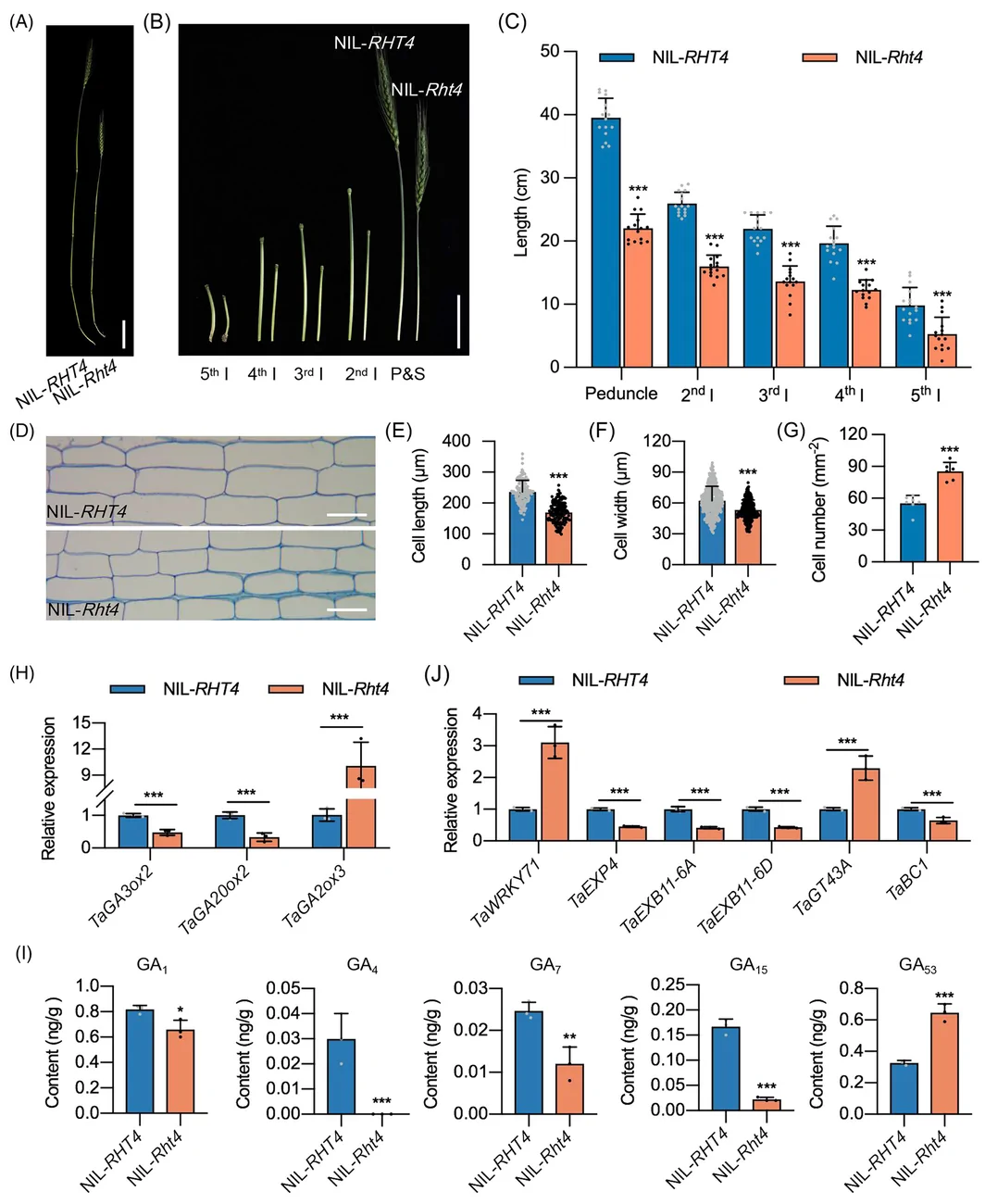
*Rht4* regulates plant height by modulating cell proliferation and expansion. (A, B) Comparison of the main culm length (A) and internode length (B) between NIL‐*RHT4* and NIL‐*Rht4*. Bars = 10 cm. (C) Statistical analysis of the length of peduncles and internodes between NIL‐*RHT4* and NIL‐*Rht4*. Data are presented as means ± SD, *n*_NIL‐*RHT4*_ = 16, *n*_NIL‐*Rht4*_ = 15. (D) Longitudinal sections of the elongated peduncles from NIL‐*RHT4* and NIL‐*Rht4* at the anthesis stage. Bars = 100 μm. (E–G) Comparison of cell length (E), cell width (F) and cell number (G) of peduncles between NIL‐*RHT4* and NIL‐*Rht4*. Data are presented as means ± SD. *n*_NIL‐*RHT4*_ = 153 and *n*_NIL‐*Rht4*_ = 161 in (E), *n*_NIL‐*RHT4*_ = 388 and *n*_NIL‐*Rht4*_ = 245 in (F), and *n* = 7 in (G). (H) Expression levels of GA biosynthesis genes in elongating peduncles of NIL‐*RHT4* and NIL‐*Rht4*. (I) Measurement of endogenous GA_1_, GA_4_, GA_7_, GA_15_ and GA_53_ in the peduncles of NIL‐*RHT4* and NIL‐*Rht4*. (J) Transcription levels of genes associated with cell growth in the peduncles of NIL‐*RHT4* and NIL‐*Rht4*. Data in (E)–(J) are presented as means ± SD, *n* = 3 in (H)‐(J). *P < 0.05, **P < 0.01, ***P < 0.001, Student's *t*‐test.

To uncover the physiological mechanism underlying *Rht4*‐mediated wheat semi‐dwarfism, we examined the length of each internode of the NIL‐*Rht4* line, and found that all four internodes as well as the peduncle of NIL‐*Rht4* were significantly shorter in comparison with the NIL‐*RHT4* (Figure 2A–C), similar to other wheat dwarfing lines such as those carrying *Rht8* or *Rht24*, which also exhibit reduced length across all internodes (Chai et al., 2022; Tian, Xia, et al., 2022). As stem elongation was determined by cell division in the intercalary meristem and subsequent cell elongation in the elongation zone (Hoshikawa, 1989), paraffin sections were prepared to reveal the effects of *Rht4* on cell growth in the internode. As shown in Figure 2(D), the NIL‐*Rht4* line showed smaller cell size with significant decreases in both cell length and cell width (Figure 2E,F) while the cell number per unit area was dramatically increased in NIL‐*Rht4* compared to that in NIL‐*RHT4* (Figure 2G), suggesting that *Rht4* predominantly inhibits stem elongation through suppressing cell expansion.

To investigate the downstream pathway(s) underlying *Rht4*‐mediated wheat semi‐dwarfism, RNA sequencing (RNA‐seq) was conducted with the elongating peduncles of NIL‐*RHT4* and NIL‐*Rht4* lines at the heading stage (Z55; Zadoks et al., 1974). In total, 2726 differentially expressed genes (DEGs) were identified in NIL‐*Rht4 vs* NIL‐*RHT4*, including 802 upregulated DEGs and 1924 downregulated DEGs (Figure S3A,B; Data S1). Genes associated with GA biosynthesis and cell wall loosening or expansion were enriched among the DEGs (Data S1), implying that the reduced stem length of NIL‐*Rht4* might be a consequence of disrupted GA homeostasis. A further RT‐qPCR analysis showed that both *TaGA20ox2* and *TaGA3ox2*, the key GA biosynthesis genes, were significantly downregulated while the key GA‐deactivation gene *TaGA2ox3* was remarkably upregulated in NIL‐*Rht4* compared to NIL‐*RHT4* (Figure 2H). Further quantification of endogenous GAs in elongating peduncles at the same developmental stage showed that the contents of bioactive GA_1_ and GA_4_ as well as GA precursors including GA_15_ and GA_24_ were significantly lower in the NIL‐*Rht4* compared with the NIL‐*RHT4* (Figure 2I; Figure S3C). Particularly, GA_4_ was almost undetectable in NIL‐*Rht4*, which is consistent with the marked reduction in *TaGA20ox2* expression and GA_15_ content, whereas some GA precursors or metabolites such as GA_53_, GA_20_, GA_8_, and GA_51_ accumulated higher in comparison with NIL‐*RHT4* (Figure 2H,I; Figure S3C). Together with the stronger GA_3_ responsiveness of NIL‐*Rht4* in coleoptile length (Figure S4A,C) and plant height (Figure S4B,D), suggesting the disequilibrium GA homeostasis in NIL‐*Rht4*. In addition to transcriptional level changes of GA biosynthesis genes (Figure 2H), genes involved in cell wall synthesis and modification, cell elongation and expansion, including *TaEXP4*, *TaEXB11‐6A*, *TaEXB11‐6D*, *TaBC1*, *TaCYCB2*, and *TaPLL* (Dong et al., 2023; Lyu et al., 2024; Zhang et al., 2025), were significantly downregulated in the peduncles of NIL‐*Rht4* compared to that of NIL‐*RHT4* (Figure 2J; Figure S3D). By contrast, the transcription levels of the growth‐repressive gene *TaWRKY71* (Qin et al., 2013), cell wall remodeling and cell division/differentiation‐related genes *TaGT43A* and *TaCOBRA5* (Li et al., 2025), and *TaPRR95* (Fu et al., 2024), as well as the nitrate transporter gene *TaNPF6‐S3* (Kumar et al., 2022), were all remarkably elevated in NIL‐*Rht4* (Figure 2J; Figure S3D). Collectively, these results indicated that *RHT4* regulates stem elongation through the coordination of GA homeostasis and transcriptional level changes of cell growth‐related genes.

### Map‐based cloning of *Rht4*

**Figure 3:**
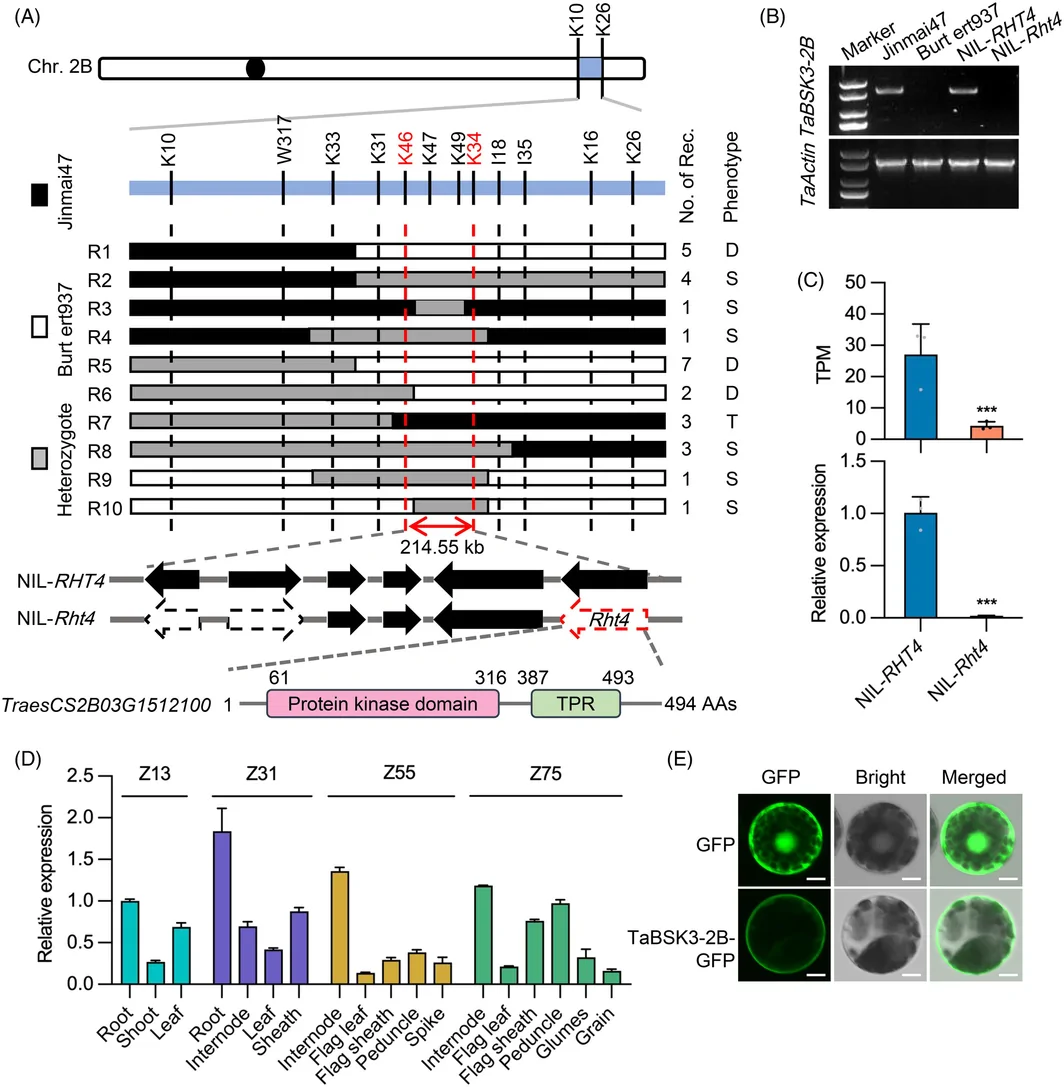
Map‐based cloning of *Rht4* and subcellular localization analysis of TaBSK3‐2B‐GFP protein. (A) Map‐based cloning of *Rht4*. *Rht4* was narrowed down to a 214.55‐kb region between molecular markers K46 and K34. D, T and S indicate dwarf (plant height ≤85 cm), tall (plant height >85 cm) and segregating (segregation for plant height) phenotypes in the progenies of the recombinant individuals. Dish‐line arrows indicate three deleted genes in the candidate region in NIL‐*Rht4* and Burt ert937 revealed by whole‐genome resequencing. (B) RT‐PCR amplification verified the deletion of *TaBSK3‐2B* in Burt ert937 and NIL‐*Rht4*. (C) Expression levels of *TaBSK3‐2B* in peduncles of NIL‐*RHT4* and NIL‐*Rht4* revealed by RNA‐seq (top panel) and RT‐qPCR (bottom panel). TPM, Transcripts Per Kilobase Million. Data are presented as means ± SD, *n* = 3. ***P < 0.001, Student's *t*‐test. (D) Relative expression of *TaBSK3‐2B* in various tissues and organs in wheat cultivar Chinese spring. Z13, three‐leaf stage. Z31, jointing stage. Z55, heading stage. Z75, grain‐filling stage. Data are presented as means ± SD, *n* = 3. (E) Subcellular localization of TaBSK3‐2B‐GFP fusion protein in wheat protoplast. Bars = 10 μm.

To clone *Rht4*, an F_2_ population with 301 individuals generated from the cross Jinmai47 × Burt ert937 was used for genetic mapping. Bulked‐segregant analysis sequencing (BSA‐seq) was combined with QTL mapping to preliminarily map *Rht4* to a ~5 Mb region between kompetitive allele‐specific PCR (KASP) marker K10 and K26 on chromosome 2BL (Figure S5A,B). Using a BC_6_F_2_ population containing 1214 individuals, *Rht4* was further narrowed down to a 214.55‐kb region flanked by molecular markers K46 and K34 (Figure 3A). Within this region, six high‐confidence genes were annotated based on the Chinese Spring (CS) reference genome (IWGSC RefSeq v.2.1) (Figure 3A). Further whole‐genome resequencing analysis identified two deleted genomic segments within the candidate region of *Rht4* in Burt ert937 and NIL‐*Rht4*. The first deletion spanned from 795 902 250 to 795 966 495 bp and encompassed *TraesCS2B03G1511100* and *TraesCS2B03G1511400*, whereas the second one spanned from 796 047 500 to 796 113 410 bp that encompassed *TraesCS2B03G1512100* (Figure 3A; Figure S6A–D). To further analyze whether there were more annotated genes in the two deleted regions, we remapped the reads to the chromosome‐scaled long‐read assembly of Jinmai47 (Jiao et al., 2025). The results showed that the same two deleted genomic segments were identified in the *Rht4* candidate interval and no additional annotated genes existed in these deleted regions (Figure S7A,B). Besides, an additional gene *JM472B883300* was identified when remapping the reads to Jinmai47 genome (Figure S7A) but was located outside the two deleted segments in the *Rht4* candidate region.

Among the three deleted genes, *TraesCS2B03G1511100* encodes a pentatricopeptide repeat‐containing protein. Its rice homolog *Os04g0684500* (*WSL5*) has been reported to be involved mainly in chloroplast development under cold stress, and the plant height and grain size of *wsl5* mutant were indistinguishable from those of wild‐type plants (Liu et al., 2018). *TraesCS2B03G1511400* encodes an amidase‐family protein with unknown function. Both genes were expressed in multiple tissues and organs of Chinese Spring, but their transcript levels were markedly reduced in the peduncles of NIL‐*Rht4* compared with NIL‐*RHT4* (Figure S8A–D; Table S4). By contrast, *TraesCS2B03G1512100* encodes a putative serine/threonine protein kinase which showed high sequence similarity with rice BSK3 (OsBSK3) (Figure 3A; Figure S9), an important BR signaling component involved in regulating plant architecture and grain size (Tian, Liu, et al., 2022; Zhang et al., 2016). RT‐PCR analysis of *TraesCS2B03G1512100* yielded bands in Jinmai47 and NIL‐*RHT4* but not in Burt ert937 or NIL‐*Rht4* (Figure 3B). This is consistent with its negligible expression in the peduncles and other organs of Burt ert937 or NIL‐*Rht4* revealed by RNA‐seq and RT‐qPCR analysis (Figure 3C; Figure S10). Therefore, *TraesCS2B03G1512100* was considered the most likely candidate gene underlying *Rht4* locus, thereafter designated as *TaBSK3‐2B*.

Sequence analysis showed that the putative TaBSK3‐2B protein is composed of 494 amino acid residues with a predicted serine/threonine protein kinase domain (E61‐Q316) and a TPR domain (D387‐G493) (Figure 3A; Figure S9). Multiple sequence alignment showed that TaBSK3‐2B shared the same conserved domains with AtBSK3 and OsBSK3 (Figure S9). Phylogenetic analysis showed that homologs of TaBSK3‐2B were ubiquitously distributed in plant species (Figure S11). Expression pattern analysis revealed that *TaBSK3‐2B* was ubiquitously expressed in various tissues and organs at three‐leaf stage (Z13) and jointing stage (Z31), and predominantly expressed in the internodes at heading stage (Z55) and grain‐filling stages (Z75) (Figure 3D; Figure S10), indicating its potential involvement in stem elongation and the control of grain size during wheat growth and development. Subcellular localization analysis of C‐terminal‐GFP‐tagged TaBSK3‐2B in wheat protoplasts indicated that TaBSK3‐2B‐GFP was predominantly localized at the plasma membrane (Figure 3E), which was consistent with its homologs in rice and *Arabidopsis* (Ren et al., 2019; Tang et al., 2008; Zhang et al., 2016).

### Functional validation of *TaBSK3‐2B* as the potential causal gene of *Rht4*

**Figure 4:**
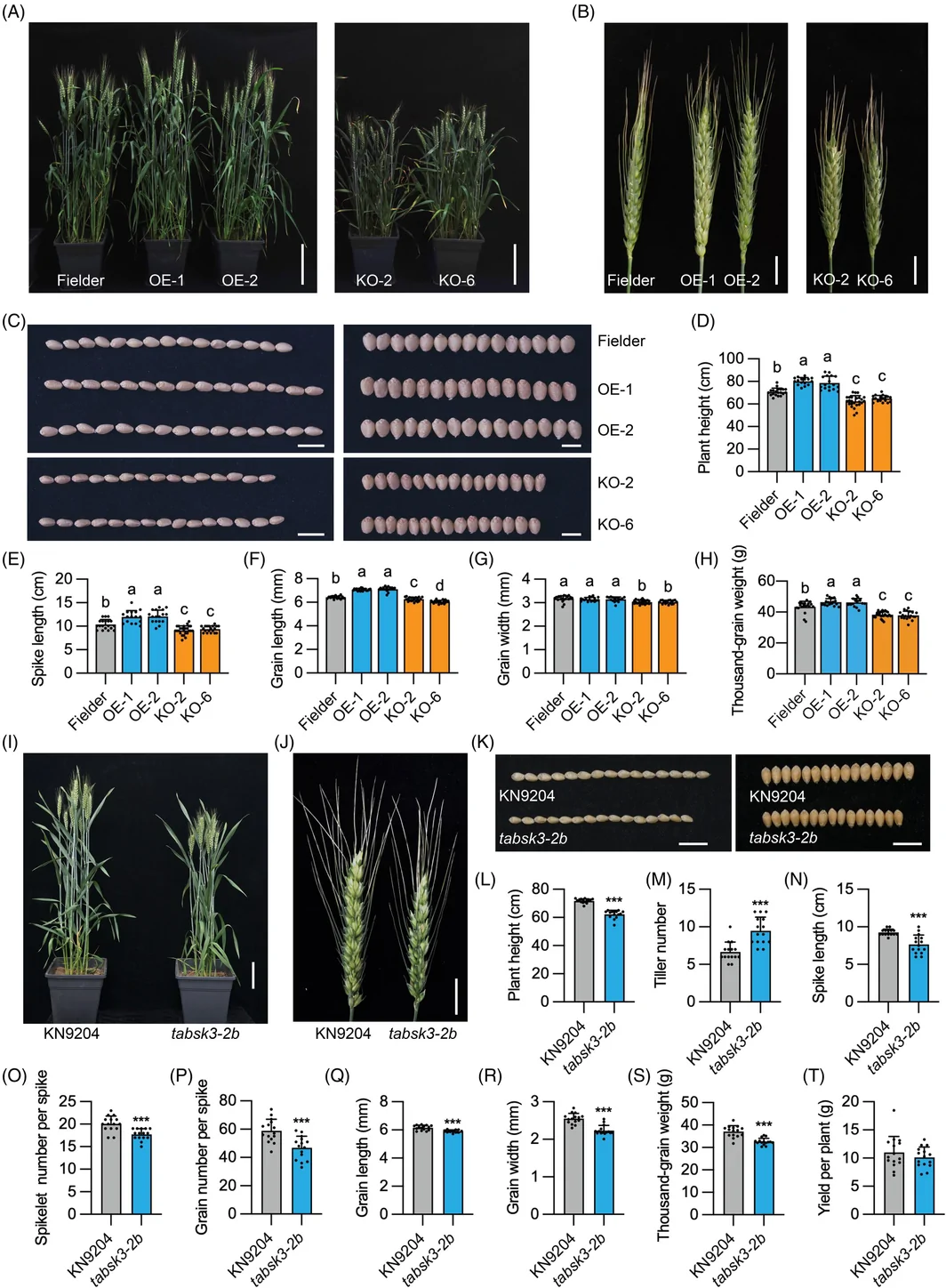
Functional confirmation of *TaBSK3‐2B*. (A) Gross morphologies of Fielder, *TaBSK3‐2B* overexpression (OE‐1, OE‐2) and *TaBSK3* gene‐editing (KO‐2, KO‐6) lines at grain‐filling stage. Bars = 5 cm. (B, C) Comparison of spike length (B) and grain size (C) of Fielder, OE‐1, OE‐2, KO‐2 and KO‐6 lines. Bars = 2.5, 1 and 0.5 cm, respectively. (D–H) Statistical analysis of plant height (D), spike length (E), grain length (F), grain width (G), thousand‐grain weight (H) of Fielder, OE‐1, OE‐2, KO‐2 and KO‐6 plants. Data are presented as means ± SD, *n* = 16–25. Different letters in (D)–(H) indicate significant differences at *P* < 0.05, one‐way ANOVA, Tukey's HSD test. (I) Gross morphologies of KN9204 (WT) and *tabsk3‐2b* at grain‐filling stage. Bar = 10 cm. (J, K) Comparison of spike length (J) and grain size (K) of KN9204 and *tabsk3‐2b* lines. Bars = 2, 1 and 1 cm, respectively. (L–T) Statistical analysis of plant height (L), tiller number (M), spike length (N), spikelet number per spike (O), grain number per spike (P), grain length (Q), grain width (R), thousand‐grain weight (S) and yield per plant (T) of KN9204 and *tabsk3‐2b*. Data are presented as means ± SD, *n* = 15. ***P < 0.001, Student's *t*‐test.

To confirm the function of *TaBSK3‐2B* in regulating wheat plant architecture, we generated multiple *TaBSK3‐2B* overexpression (OE) transgenic lines in Fielder background, and two lines OE‐1 and OE‐2 were selected for further analysis (Figure S12B). We also generated *TaBSK3‐2B* edited lines through CRISPR‐Cas9 gene editing using two guide RNAs targeting the second exon of the *TaBSK3‐2B*. In the Fielder background, two identical *TaBSK3* copies were detected on the A genome and one copy was detected on the B genome, whereas no D‐genome copy was identified. Two kinds of homozygous knockout (KO) mutants (KO‐2 and KO‐6) harboring insertions/deletions (InDels) were identified through PCR screening and sequencing analysis (Figure S12A). Compared to the wild‐type plants, the OE‐1 and OE‐2 lines showed significant increases in plant height, spike length, grain length as well as thousand‐grain weight, while the knocking out of multiple copies of *TaBSK3* led to remarkable decreases in plant height, spike length, grain size, and grain weight (Figure 4A–H). Moreover, significant increases in tiller number and grain roundness were observed in KO‐2 and KO‐6 lines, whereas the overexpression of *TaBSK3‐2B* resulted in a slight decrease in wheat tiller number and grain roundness (Figure S12C,H). Additionally, neither the *TaBSK3‐2B* overexpression lines nor the KO lines exhibited significant changes in spikelet number (Figure S12D), grain yield per plant (Figure S12F), or harvest index (Figure S12G) in comparison with the wild‐type plants.

To further test whether the functional disruption of *TaBSK3‐2B* alone is sufficient to regulate plant height, we identified an EMS mutant, *tabsk3‐2b*, carrying a premature stop codon in *TaBSK3‐2B* in the KN9204 background (Figure S13). Compared with wild‐type KN9204, *tabsk3‐2b* showed significantly reduced plant height and spike length (Figure 4I,J,L,N). Under field conditions, *tabsk3‐2b* plants were significantly shorter than the wild type with shorter spikes (Figure 4L,N) and altered yield‐related traits, including reduced spikelet number per spike (Figure 4O), grain number per spike (Figure 4P), grain size (Figure 4Q,R), and thousand‐grain weight (Figure 4S). In contrast, tiller number was significantly increased in *tabsk3‐2b* compared with the wild type (Figure 4M). Distinct from the NIL‐*Rht4* line, loss function of *TaBSK3‐2B* alone did not compromise the grain yield per plant (Figure 4T), suggesting the potential involvement of the other two deleted genes in controlling grain yield. Taken together, these results demonstrated that *TaBSK3‐2B* is a positive regulator of wheat plant height and grain size. As the CRISPR‐Cas9 engineered *tabsk3* mutants and the independent null mutant of *TaBSK3‐2B* showed semi‐dwarfism that recapitulated the NIL‐*Rht4*, it was confirmed that deletion of *TaBSK3‐2B* accounts for wheat *Rht4* semi‐dwarf phenotype.

### *TaBSK3‐2B* positively regulates BR responses in wheat

**Figure 5:**
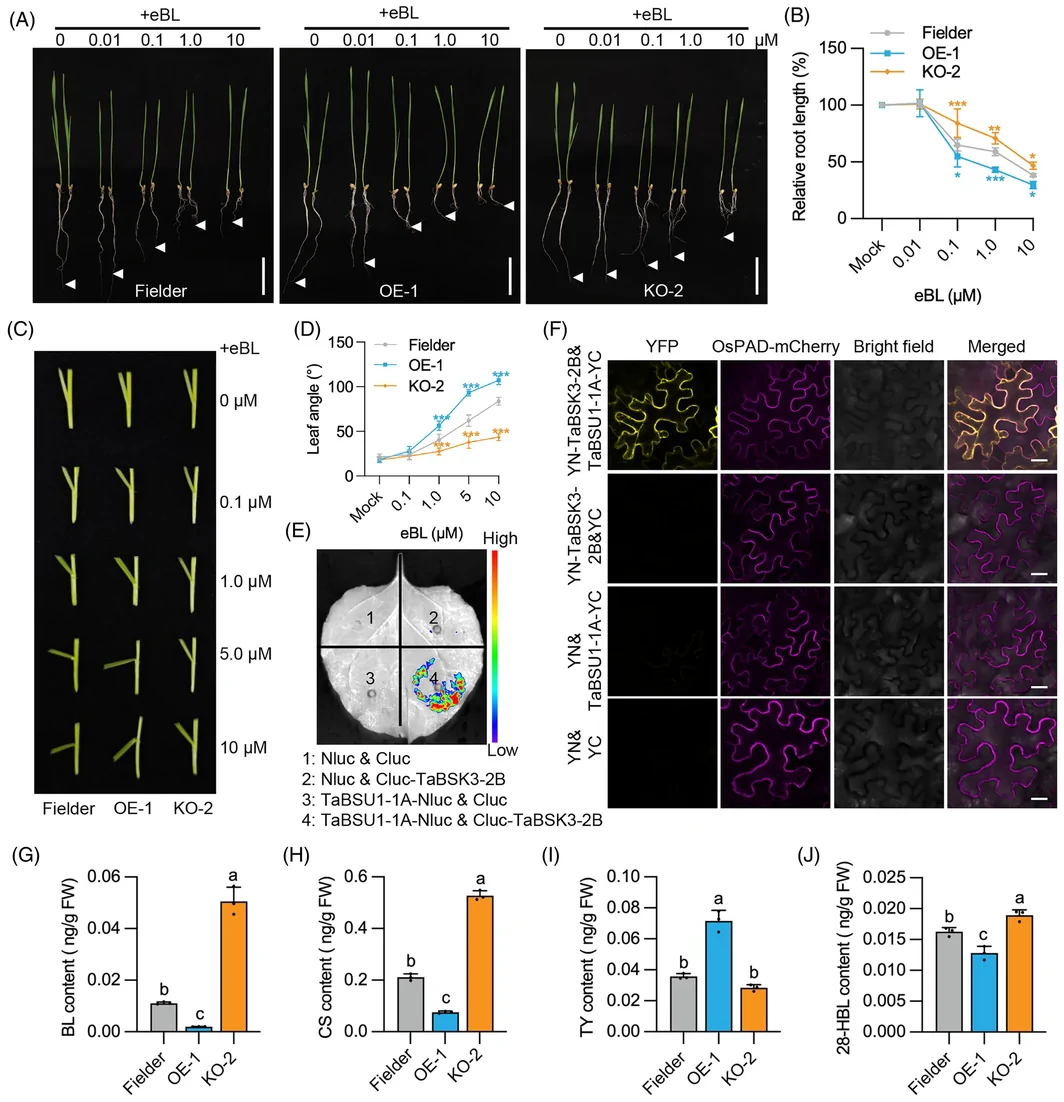
*TaBSK3‐2B* regulates wheat BR responses. (A) Root elongation assay of the Fielder, *TaBSK3‐2B* OE‐1 and *TaBSK3* KO‐2 lines in response to various concentrations of epi‐brassinolide (eBL). Bars = 5 cm. (B) Statistical analysis of the relative root length in response to exogenous eBL. (C) The lamina inclination assay of Fielder, *TaBSK3‐2B* OE‐1 and *TaBSK3* KO‐2 lines upon eBL treatment. (D) Statistical analysis of the leaf angle in response to exogenous eBL. Data are presented as means ± SD, *n* = 15. *P < 0.05, **P < 0.01, ***P < 0.001, Student's *t*‐test. (E) Firefly luciferase complementation assay in *Nicotiana benthamiana* leaves was used to detect interaction between TaBSK3‐2B and TaBSU1‐1A proteins. The Nluc and Cluc are empty vectors. (F) Bimolecular fluorescence complementation assay showing the interaction between TaBSK3‐2B and TaBSU1‐1A in *N. benthamiana* leaves. TaBSK3‐2B was fused with the N‐terminal fragment of YFP (YN), TaBSU1‐1A was fused with the C‐terminal fragment of YFP (YC). The OsPAD‐mCherry was used as a plasma membrane marker. Bars = 20 μm. (G–J) Measurement of endogenous brassinolide (BL) (G), castasterone (CS) (H), typhasterol (TY) (I) and 28‐homobrassinolide (28‐HBL) (J) in the peduncles of Fielder, *TaBSK3‐2B* OE‐1 and *TaBSK3* KO‐2 lines at heading stage. Data in (G–J) are presented as means ± SD, *n* = 3. Different letters in (G)–(J) indicate significant differences at *P* < 0.05, one‐way ANOVA, Tukey's HSD test.

Given that TaBSK3‐2B is the ortholog of the BR signaling kinase OsBSK3, we hypothesized it functions in the BR pathway. To test this, we assessed BR sensitivity in *TaBSK3‐2B* overexpression (OE) and knockout (KO) lines using exogenous epi‐brassinolide (eBL). In root elongation assays, the KO mutant showed attenuated inhibition of root length by eBL, whereas the OE line displayed enhanced, dose‐dependent inhibition (Figure 5A,B). Similar results were observed in the BR sensitivity test of NIL‐*RHT4* and NIL‐*Rht4* lines (Figure S14). Lamina inclination assays further confirmed that the KO line was less sensitive, and the OE line more sensitive to eBL than the wild type (Figure 5C,D). These altered BR responses showed similar phenotypes to those of previously known BR signaling mutants in cereals, supporting a role for TaBSK3‐2B in BR signal transduction.

BSK proteins function downstream of the BR receptor BRI1 and relay BR signals to BSU1 phosphatases in the canonical BR signaling pathway (Kim et al., 2009; Tang et al., 2008). To verify that TaBSK3‐2B is involved in BR signal transduction, we tested the interaction between TaBSK3‐2B and TaBSU1‐1A, the wheat homolog of rice BR signaling kinase BSU1. A firefly luciferase complementation imaging assay detected interaction between Cluc‐TaBSK3‐2B and TaBSU1‐1A‐Nluc in *Nicotiana benthamiana* leaves, whereas no signal was detected in the control groups (Figure 5E). This interaction was further supported by bimolecular fluorescence complementation (BiFC) assays, in which reconstituted YFP fluorescence was detected at the plasma membrane when YN‐TaBSK3‐2B and TaBSU1‐1A‐YC were co‐expressed (Figure 5F). These results support a physical interaction between TaBSK3‐2B and TaBSU1‐1A in vivo. Altered BR sensitivity often triggers feedback regulation of BR biosynthesis (Song et al., 2023; Tong et al., 2009; Yamamuro et al., 2000). We analyzed the transcription levels of BR biosynthesis genes in peduncles from *TaBSK3‐2B* OE and *TaBSK3* KO lines. Results showed that the expression levels of BR biosynthesis gene *TaD11*, *TaDWARF4*, and *TaBR6ox1* were notably changed in both OE and KO lines compared to Fielder (Figure S15). Besides, *TaBR6ox2* was also upregulated in the KO plants whereas transcription levels of *CONSTITUTIVE PHOTOMORPHOGENIC DWARF* (*TaCPD*) were not changed in *TaBSK3* KO line but upregulated in OE plants compared to that in Fielder (Figure S15). Furthermore, quantification of the endogenous BRs including brassinolide (BL), castasterone (CS), and 28‐homobrassinolide (28‐HBL) showed that all of them were significantly increased in *TaBSK3* KO line but decreased in the *TaBSK3‐2B* OE line compared to that in Fielder plants (Figure 5G,H,J), which was consistent with the negative feedback regulation in BR signaling mutants (Tong et al., 2009; Yamamuro et al., 2000). Besides, higher accumulation of typhasterol (TY) was detected in *TaBSK3‐2B* OE line (Figure 5I). Taken together, these results suggested that *TaBSK3‐2B* acts as a positive regulator of BR signaling to shape wheat plant architecture and grain yield.

### *TaBSK3‐2B* positively regulates wheat drought tolerance

**Figure 6:**
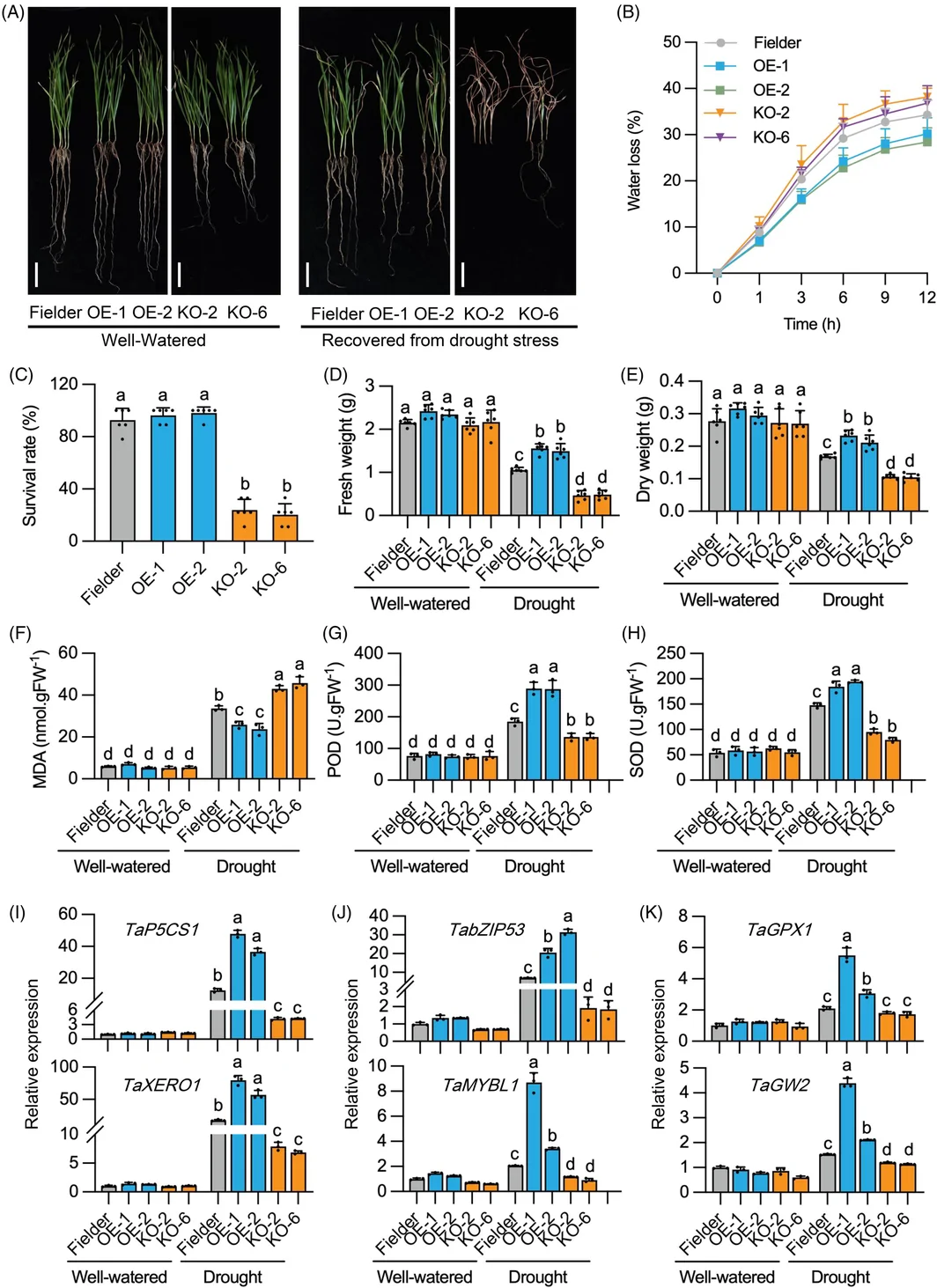
*TaBSK3‐2B* acts as a positive regulator of wheat drought tolerance. (A) Assessment of drought tolerance of Fielder, *TaBSK3‐2B* OE‐1 and OE‐2, *TaBSK3* KO‐2 and KO‐6 lines at seedling stage. Pot‐grown wheat seedlings were subjected to a 14‐day dehydration at three‐leaf stage, followed by a 7‐day period of recovery with full irrigation. Left panel, parallel control groups under well‐watered condition. Right panel, recovered wheat seedlings after dehydration‐re‐watering treatment. Bars = 5 cm. (B) Water loss rate assay of detached leaves of Fielder, *TaBSK3‐2B* OE and *TaBSK3* KO lines. (C) Survival rates of Fielder, OE and KO lines after a 7‐day recovery from dehydration treatment. (D, E) Seedlings' fresh weight (D) and dry weight (E) of lines in (A). (F–H) The contents of malondialdehyde (MDA) (F), peroxidase (POD) (G) and superoxide dismutase (SOD) (H) in leaves of WT and transgenic plants under well‐watered and drought conditions. (I–K) Relative expression levels of drought‐responsive genes in Fielder, *TaBSK3‐2B* OE and *TaBSK3* KO plants. Data are presented as means ± SD, *n* = 10 in (B), *n* = 6 in (C)–(E), *n* = 3 in (F)–(K). Different letters in (C)–(K) indicate significant differences (*P* < 0.05, one‐way ANOVA, Tukey's HSD test).

Building on prior reports that *Rht4* reduces plant height without compromising coleoptile length or seedling vigor, key traits for wheat deeper sowing in arid regions, we further examined coleoptile elongation in the *TaBSK3‐2B* overexpression and *TaBSK3* knockout lines. The coleoptile lengths of the OE‐1 and OE‐2 lines were comparable to or significantly longer than that of Fielder, whereas the knockout lines KO‐2 and KO‐6 showed significantly shorter coleoptiles (Figure S16). We next evaluated the role of *TaBSK3‐2B* in wheat drought tolerance. *TaBSK3‐2B* OE and *TaBSK3* KO lines were subjected to water‐deficit conditions (Figure 6A). A water loss rate assay at three‐leaf stage revealed that *TaBSK3* KO lines showed a significant elevation in water loss rate than Fielder, while OE plants exhibited enhanced water retention capacity in comparison with the wild type (Figure 6B). After a 14‐day dehydration treatment followed by a 7‐day recovery period with full irrigation, most of the *TaBSK3* KO plants could not recover, showing significant reductions in survival rates compared to Fielder. However, the OE‐1 and OE‐2 lines had comparable survival rates to the Fielder (Figure 6A,C). Consistently, overexpression of *TaBSK3‐2B* could significantly increase biomass including fresh weight and dry weight after dehydration treatment whereas knocking out of *TaBSK3* had opposite impacts on wheat biomass under drought conditions (Figure 6D,E). Under well‐watered conditions, there were no significant differences in the contents of malondialdehyde (MDA), peroxidase (POD) and superoxide dismutase (SOD) among *TaBSK3‐2B* OE, *TaBSK3* KO, and Fielder plants (Figure 6F–H). However, under drought stress, OE plants accumulated less MDA and exhibited higher POD and SOD activities than Fielder, whereas KO plants displayed the opposite trend (Figure 6F–H), indicating that *TaBSK3‐2B* enhances drought tolerance by mitigating oxidative damage and promoting antioxidant capacity.

To explore the molecular basis, we analyzed the expression of drought‐responsive genes by RT‐qPCR. Under drought conditions, key genes involved in proline biosynthesis (*TaP5CS1*) and dehydration response (*TaXERO1*) were upregulated in OE lines but downregulated in KO lines (Figure 6I). Similarly, transcripts of the regulatory genes *TabZIP53*, *TaMYBL1*, and *TaGW2*, as well as the oxidative stress‐related gene *TaGPX1*, were elevated in OE plants but suppressed in KO plants upon drought stress (Figure 6J,K). Collectively, these results indicate that *TaBSK3‐2B* acts as a positive regulator of drought tolerance in wheat, integrating transcriptional reprogramming of stress‐adaptive genes with the activation of physiological protective mechanisms.

### Natural variation of *TaBSK3‐2B* and its geographical distribution

**Figure 7:**
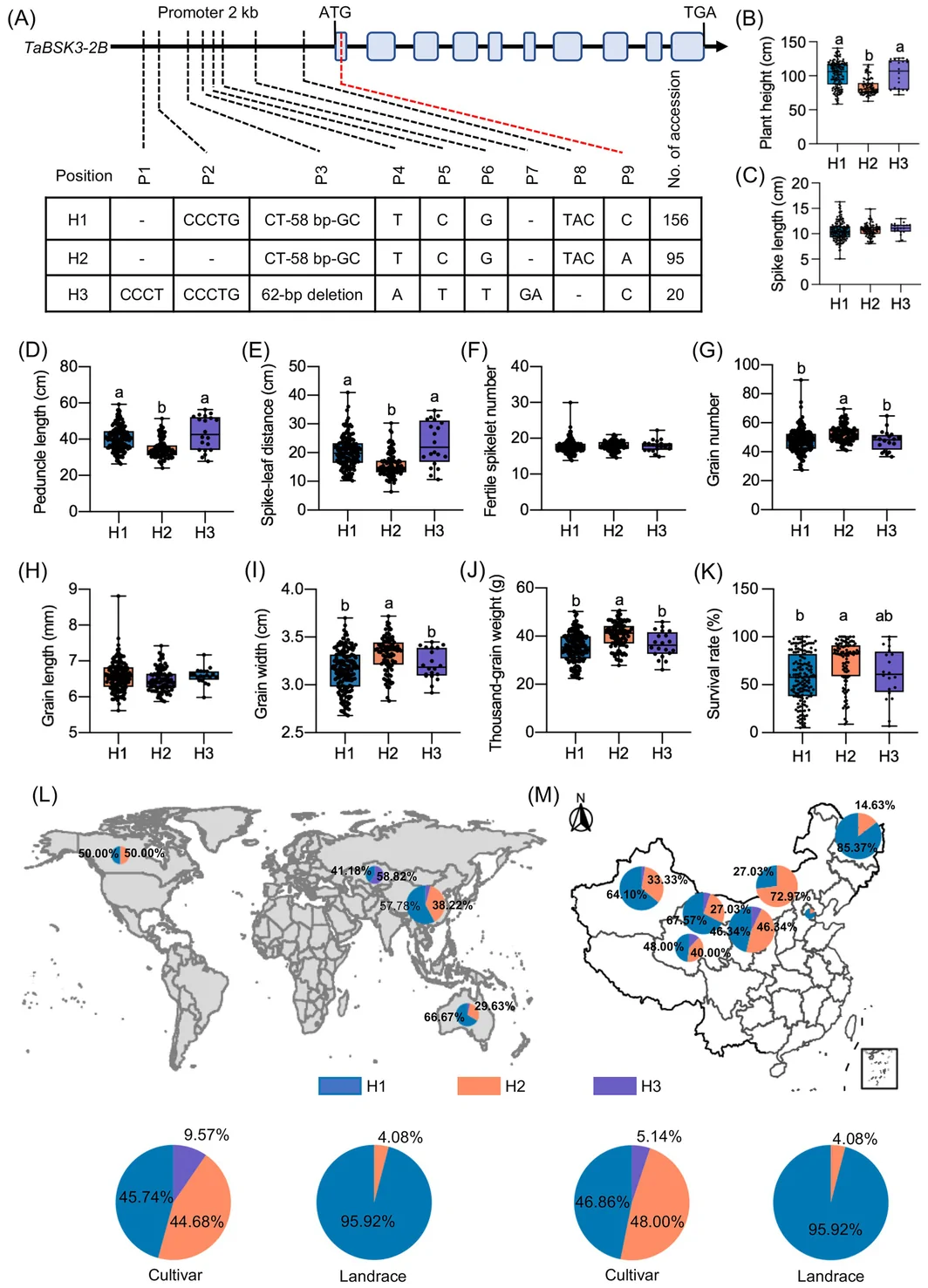
Haplotypes analysis *TaBSK3‐2B* in a natural spring wheat population. (A) Haplotype analysis of *TaBSK3‐2B* among 271 spring wheat accessions. Boxes indicate the exons of *TaBSK3‐2B*. (B–K) Comparison of plant height (B), spike length (C), peduncle length (D), spike‐leaf distance (E), fertile spikelet number (F), grain number (G), grain length (H), grain width (I), thousand‐grain weight (J) and survival rate (K) among accessions carrying three haplotypes of *TaBSK3‐2B*. Each data point represents the BLUP value of indicated agronomic traits under five planting sites within 2 years. Different letters in (B)–(K) indicate significant differences at *P* < 0.05, Kruskal–Wallis test. (L, M) Geographical distribution of three haplotypes of *TaBSK3‐2B* in the world (L, *n* = 271) and in China (M, *n* = 225).

To identify natural alleles of *TaBSK3‐2B* that may potentially contribute to high‐yield semi‐dwarf wheat breeding, we performed haplotype analysis using 271 spring wheat accessions (Table S5). Variations in the promoter region and coding sequence of *TaBSK3‐2B* were examined, and the population was classified into three haplotypes based on nine plant height‐associated polymorphisms, including SNPs and InDels in the promoter and one SNP in exon 1 (Figure 7A; Table S6). Compared to wheat accessions carrying *TaBSK3‐2B* ^*H1*^ or *TaBSK3‐2B* ^*H3*^, accessions harboring *TaBSK3‐2B* ^*H2*^ exhibited the lowest plant height, shortest peduncle length, and spike‐leaf distance (the vertical distance between the base of the spike and the leaf stalk of the flag leaf on the main stem; Figure S18) (Figure 7B,D,E), suggesting that *TaBSK3‐2B* ^*H2*^ has the strongest effects in inhibiting stem elongation thus reducing plant height. No significant differences were observed among haplotypes in spike length, fertile spikelet number, or grain length (Figure 7C,F,H). Most importantly, accessions with *TaBSK3‐2B* ^*H2*^ produced more grains with significant increases in grain width and thousand‐grain weight than those with *TaBSK3‐2B* ^*H1*^ and *TaBSK3‐2B* ^*H3*^ (Figure 7G,I,J). Moreover, no significant difference in coleoptile length was detected among the three haplotypes (Figure S17), whereas accessions carrying *TaBSK3‐2B* ^*H2*^ showed a higher seedling survival rate after drought stress than those carrying *TaBSK3‐2B* ^*H1*^ and comparable to those carrying *TaBSK3‐2B* ^*H3*^ (Figure 7K), demonstrating its potential in wheat semi‐dwarf and drought‐resilient breeding. Haplotype frequency analysis indicated that *TaBSK3‐2B* ^*H2*^ was distributed in 44.68% of the worldwide cultivars and 48% of the Chinese cultivars in the spring wheat panel, while only 4.08% of the landraces in these accessions were identified with *TaBSK3‐2B* ^*H2*^ (Figure 7L,M). Although the distribution of *TaBSK3‐2B* ^*H2*^ varies in major growing regions of spring wheat, ranging from 14.63 to 72.97%, the significant elevation of *TaBSK3‐2B* ^*H2*^ frequency in spring wheat cultivars compared to landraces indicated a strong artificial selection of *TaBSK3‐2B* ^*H2*^ in spring wheat breeding practices. Taken together, these results identified *TaBSK3‐2B* ^*H2*^ as a naturally occurring haplotype associated with reduced plant height and improved yield traits. Future evaluation of the *TaBSK3*‐*2B* ^*H2*^ in NILs would be informative for its potential application in breeding new semi‐dwarf wheat varieties without penalties of grain traits.

## DISCUSSION

The introduction of Green Revolution alleles into breeding programs unprecedentedly increased global cereal production. Nevertheless, the GA‐insensitive *Rht‐B1b* and *Rht‐D1b* compromise wheat coleoptile length, seedling establishment, grain weight, nitrogen use efficiency, and resistance to biotic and abiotic stresses (Jatayev et al., 2020; Li et al., 2006; Rebetzke et al., 1999, 2007; Srinivasachary et al., 2009; Zhao et al., 2022). A deeper understanding of the molecular basis underlying wheat plant height would substantially benefit the development of environment‐friendly semi‐dwarf varieties and sustainable crop production. In this study, we cloned the *TaBSK3‐2B* as the potential causal gene of the *Rht4* semi‐dwarf locus and found that *tabsk3‐2b* attenuates wheat BR signaling to regulate wheat plant architecture and drought tolerance. We also identified *TaBSK3‐2B* ^*H2*^ in a spring wheat panel as a natural allele that may balance the trade‐off between plant height and grain traits (Figure 7) to facilitate its potential application in breeding new semi‐dwarf wheat varieties.

### Attenuation of TaBSK3‐2B‐mediated BR signaling contributes to the pleiotropic effects of *Rht4* on wheat growth and development

Although the GA‐sensitive *Rht4* was considered a promising semi‐dwarfing gene for breeding drought‐tolerant wheat varieties under limited water condition (Du et al., 2018), the genetic structure underlying *Rht4* locus has long been unclear. Using map‐based cloning, we characterized *TaBSK3‐2B*, the ortholog of rice *OsBSK3*, as the major causal gene of *Rht4* (Figure 3; Figure S9). In rice and *Arabidopsis*, BSK3 kinases act as BR signaling component to regulate plant growth and development by promoting BR signal transduction at the plasma membrane (Ren et al., 2019; Tian, Liu, et al., 2022; Zhang et al., 2016). In this study, we demonstrated that the deletion of *TaBSK3‐2B* underlies the pleiotropic effects of *Rht4* semi‐dwarf locus by modulating BR signaling. Disrupting the function of *TaBSK3‐2B* remarkably reduced plant height and grain size, whereas overexpression of *TaBSK3‐2B* had positive effects on wheat growth and development (Figure 4A,C,D,H,I,Q–S). Meanwhile, BR sensitivity of *TaBSK3* KO line was decreased compared to Fielder, whereas OE plants were more sensitive in response to eBL treatment (Figure 5A–D), supporting the notion that TaBSK3‐2B functions as a positive regulator in the BR signaling pathway and thereby controls wheat plant height and grain yield. It was demonstrated that impaired BR signaling could induce compensatory upregulation of BR biosynthetic genes and repression of BR deactivation genes, leading to endogenous BR accumulation (Niu et al., 2022; Wang et al., 2012). Our results showed that the levels of endogenous BL, CS, and 28‐HBL were significantly increased in *TaBSK3* KO plants (Figure 5G,H,J). These increases were likely attributed to the upregulation of BR biosynthesis genes, including *TaD11*, *TaDWARF4*, and *TaBR6ox1* in these lines (Figure S15).

The *Rht4* allele in Burt ert937 carries two large‐fragment deletions removing three genes within the candidate interval (Figure S6; Table S4). In addition to *TaBSK3‐2B*, *TaWSL5* and *TraesCS2B03G1511400* were also deleted at the *Rht4* locus (Figure S6; Table S4). The rice homolog of the *TaWSL5* mainly functions in chloroplast development under cold stress, and its mutant shows no change in plant height or grain size (Liu et al., 2018), whereas the function of the homologs of the deleted amidase‐family gene (*TraesCS2B03G1511400*) remains unclear in crops. Given that either disruption of *TaBSK3* by CRISPR/Cas9 technology or independent EMS‐mutagenized *tabsk3‐2b* mutant could reproduce the semi‐dwarfism of the *Rht4*, together with the fact that *TaBSK3‐2B* overexpression increased plant height (Figure 4; Figure S13), the other two genes are unlikely to play major roles in regulating wheat plant height. Nevertheless, *TaBSK3‐2B* disruption may not explain all effects of the original *Rht4* allele. NIL‐*Rht4* showed a significant grain‐yield penalty (Figure 1), whereas *TaBSK3* KO lines and *tabsk3‐2b* mutant showed no significant change in grain yield per plant compared with the corresponding wild‐type plants (Figure 4; Figure S12). This difference suggested that the yield penalty of NIL‐*Rht4* may be partially attributed to the deletion of the other two genes or the differences in genetic background. Thus, alleles that modulate *TaBSK3‐2B* without deletion of other genes at *RHT4* locus, such as *TaBSK3‐2B* ^*H2*^, may offer a better resource to coordinate plant height and yield traits in wheat breeding under the condition of future evaluation.

In addition, we found that *TaBSK3‐2B* plays an essential role in wheat drought tolerance. The enhanced drought tolerance of *TaBSK3‐2B* OE lines could be ascribed to the transcriptional level changes of multiple drought‐inducible genes and physiological adjustments, including increased activity of SOD and POD and decreased MDA accumulation under drought conditions (Figure 6f–k). In wheat, *TaBZR2* could directly bind to the promoter of *glutathione s‐transferase‐1* (*TaGST1*) to activate *TaGST1* expression and enhance superoxide anions ($O_{2}^{-}$) scavenging to improve drought tolerance (Cui et al., 2019), suggesting a crosstalk between BR signaling and drought tolerance. Consistently, *TaBSK3* KO lines with impaired BR sensitivity were more susceptible to dehydration treatment (Figure 6A,B). It should be noted that these drought tolerance assays were performed under controlled environmental conditions; further evaluation under field conditions will be essential to assess the agronomic application value of *TaBSK3‐2B* in breeding practice. Together, our results characterized *RHT4* as a positive regulator of wheat BR signaling with pleiotropic effects on shaping wheat plant architecture and improving drought tolerance.

### RHT4 modulates BR response and GA homeostasis to regulate wheat plant height

Plant height is primarily determined by the cumulative elongation of stem internodes. At the cellular level, internode elongation is coordinated by cell proliferation in intercalary meristems and subsequent cell expansion (Gasperini et al., 2012). In wheat, *Rht8*, *Rht12*, and *Rht24* were associated with reduced internode length, which primarily resulted from decreased cell elongation (Chai et al., 2022; Sun et al., 2019; Tian et al., 2017; Xiong et al., 2022), whereas *Rht5* and *Rht14* caused semi‐dwarfism via reduced cell number in the internodes (Cui et al., 2022; Duan et al., 2022). There were no changes in the number of internodes in the *Rht4* dwarf lines in our study (Figure 2A,B). Instead, the cell number of NIL‐*Rht4* peduncles was increased while cell length in NIL‐*Rht4* was significantly shorter than that of NIL‐*RHT4*, indicating a role of *RHT4* in coordinating cell proliferation and cell expansion during stem elongation.

It has been reported that attenuation of BR signaling could suppress cell wall loosening and related elongation programs, resulting in shorter internodes and dwarfism (Nolan et al., 2020; Yin et al., 2025). Over the past decades, studies have revealed that BR promotes cell elongation via a complex signaling cascade that modulates the transcriptional regulation of growth‐related genes and crosstalk with GAs (Li et al., 2012; Nolan et al., 2020; Sun et al., 2010). In our study, we found that transcription levels of multiple cell growth‐related genes were significantly altered in *Rht4* NILs, which was consistent with inhibited cell expansion in the stem resulting from impaired BR sensitivity (Figure 2H,J; Figure S3D). BR signaling has been shown to enhance the expression of GA biosynthesis genes, including *GA20ox* and *GA3ox*, via BZR1/BES1‐dependent transcriptional regulation, thereby increasing the accumulation of bioactive GAs (Tong et al., 2014). Our results showed that the contents of endogenous GA_1_ and GA_4_ were markedly reduced in NIL‐*Rht4*, resulting from the significant downregulation of the GA‐biosynthetic genes *TaGA20ox2* and *TaGA3ox2* and upregulation of the GA‐deactivation gene *TaGA2ox3* (Figure 2H,I), reminiscent of the previously identified wheat haploblock *r*‐*e*‐*z* deletion, which confers semi‐dwarfism through the interplay of BR signaling and GA pathway (Song et al., 2023). A recent study demonstrated that the BR signaling component GSK3 can phosphorylate Rht‐B1b to enhance its inhibitory effects on downstream transcriptional factors (Dong et al., 2023), revealing a GA‐BR interaction in controlling wheat plant architecture. We propose that *RHT4* regulates wheat plant height by affecting BR responsiveness and GA homeostasis. However, it remains unclear whether *TaBSK3‐2B* directly phosphorylates GA signaling components, and future identification of its direct targets will be important for understanding how *TaBSK3‐2B* modulates GA‐related stem elongation.

### Natural variation of *TaBSK3‐2B* and its potential application in wheat semi‐dwarf breeding

Although ‘Green Revolution’ varieties with GA‐insensitive semi‐dwarfing *Rht‐B1b* and *Rht‐D1b* alleles have greatly increased grain yield by improving lodging resistance, these varieties require sufficient water and high fertilizer inputs to achieve high‐yield potential (Rebetzke et al., 1999, 2007). Due to their negative effects on early seedling establishment, the application of GA‐insensitive *Rht‐B1b* and *Rht‐D1b* alleles was less successful in arid and semi‐arid regions where deeper sowing is an important cultivation measure to overcome the low soil moisture in dry environments (Jatayev et al., 2020; Rebetzke et al., 2007; Zhao et al., 2022). In this case, GA‐sensitive dwarfing genes such as *Rht4*, *Rht8*, *Rht13*, and *Rht24* without adverse effects on coleoptile length and seedling vigor have been considered as the alternative semi‐dwarfing genes for breeding high‐yield wheat cultivars adapted to water‐limited conditions.

Since *Rht4* has been reported in 1976 (Konzak, 1976), its application in breeding practice was constrained for a long time by its unfavorable pleiotropic effects on grain traits (Du et al., 2018; Liu et al., 2017). The yield penalty associated with the original *Rht4* line (Burt ert937) might be attributed to the complete deletion of *TaBSK3‐2B* together with two neighbor genes (Figure 3A; Figures S6 and S7), raising the urgent demand of *TaBSK3‐2B* natural allele for its application in breeding new semi‐dwarf wheat varieties. We conducted a haplotype analysis of *TaBSK3‐2B* in a global panel of 271 spring wheat accessions and identified *TaBSK3‐2B* ^*H2*^ as a candidate favorable allele (Figure 7). Wheat accessions with *TaBSK3‐2B* ^*H2*^ not only exhibited reduced plant height but also were associated with increased grain number, grain width, and grain weight than those with *TaBSK3‐2B* ^*H1*^ or *TaBSK3‐2B* ^*H3*^ haplotypes (Figure 7G–I). The dramatic increase of *TaBSK3‐2B* ^*H2*^ allele frequency (4.08% in landraces versus >44% in cultivars) in commercial released varieties (Figure 7L,M) suggested that this haplotype may have undergone artificial selection in wheat breeding practice. Nevertheless, the population‐level association does not establish *TaBSK3‐2B* ^*H2*^ as the causal variant underlying these traits. Future functional validation in near‐isogenic background, together with analyses of the mechanism of action of *TaBSK3‐2B* ^*H2*^, will be required to define the molecular basis and its breeding value in synergistic improvement of wheat plant height and grain yield. As *TaBSK3‐2B* acts as a positive regulator of wheat drought tolerance (Figure 6), further evaluation of the performance of *TaBSK3‐2B* ^*H2*^ under limited water condition would be more informative for its potential use in genetic improvement of wheat drought tolerance. Although two studies have indicated that pyramiding of *Rht4* with *Rht8* or *Rht‐B1b* could compromise some adverse effects of original *Rht4* on grain yield, this limited improvement is largely dependent on the genetic background of parental lines (Du et al., 2018; Liu et al., 2017). *TaBSK3‐2B* ^*H2*^ may represent a more promising candidate haplotype for developing resilient semi‐dwarf varieties compared with previous strategies relied on compensatory gene pyramiding. Understanding the contribution of *TaBSK3‐2B* ^*H2*^ to plant architecture, yield‐related traits and stress adaptation provides new insights into the coordination of plant height and grain yield in breeding high‐yield wheat varieties through modulation of BR signaling pathway.

## MATERIALS AND METHODS

### Plant materials and growth condition

Wheat variety Jinmai47 and Burt ert937 were used as parents for NIL development, which were planted at the experimental farm of Northwest A&F University (Shaanxi, China). The *tabsk3‐2b* EMS mutant was obtained from the KN9204 EMS mutant library (Wang et al., 2023), whose mutation was confirmed by PCR amplification and Sanger sequencing. *TaBSK3‐2B* overexpression and CRISPR/Cas9 gene‐edited lines were generated in Fielder background and grown in the greenhouse. The temperature and photoperiod for wheat in‐house grown were 24°C day/20°C night and 16‐h light/8‐h dark with light illumination ~3000 lux. Tobacco (*N. benthamiana*) plants were grown in a growth chamber at 23°C under a 16‐h light/8‐h dark cycle for 4–5 weeks before infiltration.

### Phenotype evaluation

At maturity stage (Z91, Zadoks et al., 1974), plant height, spike length, length of peduncles, and first (1st) to forth (4th) internode length from the soil surface to the top of the ear (excluding awns) of the main tiller were measured at physiological maturity. Main culm of individual plants were used to investigate the spikelet number per spike, grain number per spike, grain yield per plant and aboveground biomass per plant. Harvest index was calculated as grain yield per plant divided by aboveground biomass per plant. Wheat kernels were harvested individually for determination of thousand‐grain weight, grain length, grain width, and grain roundness, which were analyzed by a Crop Grain Appearance Quality Scanning Machine (SC‐E, Wanshen Technology Company, Hangzhou, China).

### Cell size analyses

Paraffin sections were performed according to previously described (Chai et al., 2022). Briefly, one‐centimeter sections in the middle of peduncles from wheat plant individuals were collected at the anthesis stage (Z65; Zadoks et al., 1974) and fixed in FAA solution at 4°C for 12 h. Longitudinal sections were prepared and stained with toluidine blue. Images were captured using a ZEISS Primo Star microscope (ZEISS, Germany). Cell length, width, and number were quantified using ImageJ (https://imagej.nih.gov/ij/index.html).

### RNA sequencing

Total RNA was extracted from elongating peduncles of Jinmai47, Burt ert937, NIL‐*RHT4*, and NIL‐*Rht4* plants at heading stage (Z55; Zadoks et al., 1974). Library construction and sequencing were performed by Novogene Corporation (Beijing, China). RNA‐seq data were generated using the Illumina HiSeq 4000 platform. Fastp was used to filter reads and correct the raw read data. Clean reads from each library were aligned to the wheat reference genome (IWGSC RefSeq v2.1) using HISAT2 (v2.0.5) (Kim et al., 2019). Transcription‐level quantification was performed with FeatureCounts (v1.5.0‐p3). Differentially expressed genes (DEGs) were identified using the DESeq2 R package (v1.20.0) (Love et al., 2014), treating each sample as a distinct factor. Genes with Log2 fold change ≥1 and false discovery rate (FDR) <0.05 were considered significantly differentially expressed.

### Real‐time quantitative PCR

Total RNA was extracted from elongating peduncles (Z55; Zadoks et al., 1974) using RNAiso Plus (TaKaRa, Shiga, Japan) and first‐strand cDNA was synthesized with the iScience RT Reagent Kit (EG15133S, iScience, Jiangsu, China). The real‐time quantitative PCR (RT‐qPCR) was performed with Taq‐HS SYBR® Green qPCR Premix (EG20113M, iScience, Jiangsu, China) on the Applied Biosystems™ QuantStudio™ 3 (Thermo Fisher Scientific, USA). Gene expression levels were normalized to the *TaActin* using the 2^−ΔΔCt^ method (Livak & Schmittgen, 2001). Primers used for RT‐qPCR are listed in Table S1.

### Quantification of endogenous gibberellins (GAs) and brassinosteroids (BRs)

Peduncles of wheat plants were collected at elongation stage (Z55; Zadoks et al., 1974) and endogenous GAs (GA_1_, GA_3_, GA_4_, GA_7_, GA_5_, GA_6_, GA_8_, GA_9_, GA_13_, GA_15_, GA_19_, GA_20_, GA_24_, GA_34_, GA_44_, GA_51_) and BRs (brassinolide, castasterone, 28‐homobrassinolide, typhasterol) were measured with ESI‐HPLC‐MS/MS by Nanjing Ruiyuan Biotechnology Co., Ltd (Nanjing, China). Three biological replicates were determined for each component.

### Genetic mapping of *Rht4*

An F_2_ population comprising 301 individuals derived from Jinmai47× Burt ert937 was used for bulked‐segregant sequencing analysis (BSA‐seq) and QTL mapping. Twenty‐five extremely tall and extremely dwarf individuals from the F_2_ segregating population were pooled for BSA‐seq, respectively. Meanwhile, 301 F_2_ plants were genotyped to construct a linkage map using R/qtl v1.74 (Broman et al., 2003) (Data S2). Kompetitive allele‐specific PCR (KASP) marker K10 and K26 were used to genotype the *Rht4* locus in F_1_ plants, and heterozygous individuals were consecutively backcrossed with Jinmai47 for six generations (BC_6_F_1_), followed by self‐pollination to develop NIL‐*RHT4* and NIL‐*Rht4*. For fine mapping, the BC_6_F_2_ population with 1214 individuals was further genotyped with KASP and insertions/deletions (InDels) markers that were developed based on sequence variations between Jinmai47 and Burt ert937. Primers used for mapping are listed in Table S2.

### Whole‐genome resequencing

Whole‐genome resequencing was performed using the DNBSEQ‐T7 platform with an average sequencing depth of approximately 20× for the parental lines, and 10× for NILs and each bulk. Low‐quality reads and adapters were trimmed using fastp (v0.23.4) (Chen et al., 2018). Clean reads were aligned to the *Triticum aestivum* reference genome (Chinese Spring v2.1) and Jinmai47 assembly (Jiao et al., 2025; Zhu et al., 2021) using BWA‐MEM (v0.7.17) (Li & Durbin, 2009), and duplicated reads were marked using Picard (v2.27.4, https://github.com/broadinstitute/picard). Variant calling was conducted using GATK (v4.5.0) (McKenna et al., 2010) following the best practice workflow. HaplotypeCaller was used to generate individual GVCF files, which were jointly genotyped using GenotypeGVCFs. Only high‐quality biallelic SNPs were retained after filtering sites with read depth <5, missing rate >20%, or minor allele frequency <0.05. The SNP‐index and ΔSNP‐index were calculated using a custom Python script (https://github.com/Lucylalala/BSAanalysis). The SNP‐index was defined as the proportion of reads supporting the alternative alleles in each bulk and ΔSNP‐index represented the absolute difference between the two bulks. A sliding‐window analysis (1–10 Mb, 0.5 Mb step) was applied to smooth ΔSNP‐index values and identified candidate genomic regions associated with the target trait. Visualization and subsequent analyses were performed in Python 3.10 using pandas, NumPy, matplotlib, and seaborn. The sequence or locus polymorphism was visualized with Integrative Genomics Viewer (IGV) v2.14.1 (Robinson et al., 2011).

### Subcellular localization analysis

The full‐length coding sequence (CDS) of *TaBSK3‐2B* was amplified and cloned into the p16318h‐GFP vectors using MonClone™ Hi‐Fusion Cloning Mix V2 (MC40101, Monada, Wuhan, China). The p16318h‐TaBSK3‐2B‐GFP plasmids were transformed into Fielder protoplasts via PEG‐mediated transformation (Yoo et al., 2007). After incubation in the dark at 25°C for 16 h, fluorescence signals were examined with the FV3000 confocal laser‐scanning microscope (Olympus, Tokyo, Japan) at excitation wavelength of 488 nm for GFP.

### Generation of transgenic lines

The CDS of *TaBSK3‐2B* was cloned into pCUB‐3 × Flag vector by MonClone™ Hi‐Fusion Cloning Mix V2 (MC40101, Monada, Wuhan, China) to generate OE lines. For generation of *TaBSK3* KO lines, two single guide RNAs (sgRNAs) targeting exon 2 of *TaBSK3‐2B* were designed by the online software E‐CRISPR (http://www.e‐crisp.org/E‐CRISP/) and cloned into pYLCRISPR/Cas9Pubi‐B plasmid as described previously (Zhang et al., 2018). All plasmids were transformed into the *Agrobacterium tumefaciens* strain EHA105 and transformed into wheat cv. Fielder according to established protocol (Ishida et al., 2015). Primer sequences are listed in Table S3.

### BR treatment

Seeds were surface sterilized and then germinated at 25°C for 2 days. Uniformly germinated wheat seeds were sown into 96‐well plates without well bottoms and immersed in Hoagland nutrient solution supplemented with 0, 0.01, 0.1, 1, and 10 μM eBL (E1641, Sigma), respectively. Root length was measured 7 days after treatment. For lamina inclination assay, two microliters of eBL solution with indicated concentrations (0, 0.1, 1, 5, or 10 μM) were applied to the top of the lamina of five‐day‐old seedlings (Fujioka et al., 1998). Images were photographed after 3‐day treatment, and the angles between the leaf blade and leaf sheath were measured using ImageJ software (https://imagej.nih.gov/ij/index.html).

### GA treatment

To evaluate GA sensitivity at the seedling stage, seeds were surface sterilized and germinated on moist filter paper. Uniformly germinated seeds were transferred to 96‐well plates without well bottoms filled with Hoagland nutrient solution supplemented with 100 μM GA3 (G8910, Solarbio) and grown in the dark at 22°C. After 7 days of treatment, coleoptile length was measured. For the plant height assay, plants were grown under field conditions and sprayed with GA3 solution (100 μM) at jointing stage (Z31, Zadoks et al., 1974), applied three times at 5‐day intervals. At maturity, plant height was measured from the soil surface to the top of the spike, excluding awns.

### Firefly luciferase complementation imaging (LCI) assay

The LCI assay was performed using the NLuc‐BS and CLuc‐BS vectors described by Luo et al. (2026). The full‐length coding sequences of TaBSK3‐2B and TaBSU1‐1A were amplified and cloned into the corresponding split‐luciferase vectors to generate Cluc‐TaBSK3‐2B and TaBSU1‐1A‐Nluc constructs. The recombinant plasmids and the corresponding empty‐vector controls were introduced into *A. tumefaciens* strain GV3101 and infiltrated into leaves of 4‐week‐old *N. benthamiana*. After 2–3 days of transient expression, luciferase signals were detected by PlantView100 (Promega). Primers used for plasmid construction are listed in Table S3.

### Bimolecular fluorescence complementation (BiFC) assay

The full‐length coding sequences of TaBSK3‐2B and TaBSU1‐1A were cloned into vectors containing the N‐ or C‐terminal fragment of yellow fluorescent protein to generate YN‐TaBSK3‐2B and TaBSU1‐1A‐YC. YN‐TaBSK3‐2B and TaBSU1‐1A‐YC fusion proteins were co‐infiltrated into 4‐week‐old *N. benthamiana* leaves using *A. tumefaciens‐mediated* transformation method (Sparkes et al., 2006). Microscopy was conducted 48 h post infiltration at the wavelengths of 514 nm for YFP and 561 nm for mCherry (FV3000, Olympus, Tokyo, Japan). Primers used for plasmid construction are listed in Table S3.

### Drought tolerance evaluation

*TaBSK3‐2B* OE, *TaBSK3* KO, and Fielder plants were grown in pots filled with a soil‐to‐vermiculite mixture (3:1) in a greenhouse. For drought treatment, water supply was withheld at the three‐leaf stage for 14 days until notable wilting was observed, followed by re‐watering for a 7‐day period of recovery. Shoot fresh weight and dry weight were recorded after 14‐day drought treatment. Seedling survival rates were assessed after recovery. Water loss assay was conducted at the three‐leaf stage using seedlings grown under normal condition. Briefly, the second fully expanded leaves were detached and immediately weighed (*W*_0_), then kept at room temperature and reweighed at defined intervals (*W*_t_). Water loss rate was calculated as (*W*_0_ − *W*_t_)/*W*_0_ × 100% and reported as the percentage of initial fresh weight lost at each time point.

The malondialdehyde (MDA), peroxidase (POD), and superoxide dismutase (SOD) contents in *TaBSK3‐2B* OE, *TaBSK3* KO, and Fielder plants were detected using the MICHY BIOLOGY assay kits (M0106A for MDA, M0105A for POD, and M0102A for SOD, Michy, Jiangsu, China) according to the manufacturer's instructions. Three biological replicates were analyzed for each experiment.

## ACCESSION NUMBERS

RNA‐seq data described in this study can be found in the NCBI Sequence Read Archive (BioProject: PRJNA1394450). Gene sequences in this study are available under the accession numbers in Table S7.

## AUTHOR CONTRIBUTIONS

QL designed the research, performed most experiments with helps from ZW, RT, YQ, XK, ZZ, CC, and YjH. SL performed data analysis of BSA‐seq and RNA‐seq. SL and YM contributed to haplotype analysis. QL, SL, YH, and LH analyzed the data and wrote the manuscript. LH, YH, and LC conceived the study, supervised the project. All the authors read and approved the manuscript.

## CONFLICT OF INTEREST

The authors declare no conflict of interest.

## Supporting information


**Data S1.** Differentially expressed genes (DEGs) identified between NIL‐*RHT4* and NIL‐*Rht4* lines.

**Data S2.** Genotypes and plant height of F_2_ individuals.

**Figure S1.** Grain roundness of NIL‐*RHT4* and NIL‐*Rht4*.
**Figure S2.** Coleoptile length of NIL‐*RHT4* and NIL‐*Rht4*.
**Figure S3.** Transcriptome analysis and gibberellins (GAs) content measurement of NIL*‐RHT4* and NIL*‐Rht4* lines.
**Figure S4.** Response of NIL‐*RHT4* and NIL‐*Rht4* to exogenous GA_3_ treatment.
**Figure S5.** Preliminary mapping of *Rht4* using bulked‐segregant analysis sequencing (BSA‐seq) and quantitative trait loci (QTL) mapping.
**Figure S6.** Whole‐genome resequencing revealed gene deletions in the dwarf NIL*‐Rht4* line and parental Burt ert937 in comparison with NIL*‐RHT4* and Jinmai47.
**Figure S7.** Whole‐genome resequencing read‐depth analysis of the *Rht4* candidate region.
**Figure S8.** Expression patterns of *TraesCS2B03G1511100* and *TraesCS2B03G1511400*.
**Figure S9.** Multiple sequence alignment of BSK3 and its homologs across plant species.
**Figure S10.** Expression patterns of *TaBSK3* in various tissues and organs in NIL*‐RHT4* and NIL*‐Rht4*.
**Figure S11.** Phylogenetic analysis of BSK proteins across plant species.
**Figure S12.** Molecular and phenotypic characterization of *TaBSK3‐2B* overexpression (OE) lines and *TaBSK3* CRISPR‐Cas9 engineered knockout (KO) mutants.
**Figure S13.** Sequence identification of *tabsk3‐2b* mutant in KN9204 background.
**Figure S14.** The growth responses of NIL*‐RHT4* and NIL*‐Rht4* seedlings upon eBL treatment.
**Figure S15.** Expression patterns of BR biosynthesis‐related genes in Fielder, *TaBSK3*‐*2B* OE‐1 and *TaBSK3* KO‐2.
**Figure S16.** Coleoptile length of *TaBSK3‐2B* overexpression (OE) and *TaBSK3* knockout (KO) lines.
**Figure S17.** Coleoptile length of accessions carrying three haplotypes of *TaBSK3‐2B*.
**Figure S18.** Definition of the spike‐leaf distance in wheat.

**Table S1.** Primers used for RT‐qPCR assay.
**Table S2.** Primers used for map‐based cloning.
**Table S3.** Primers used for plasmid construction and genotyping.
**Table S4.** Information of the three deleted genes within the mapping interval.
**Table S5.** Information of 271 spring wheat accessions.
**Table S6.** Sequence variations of the three major haplotypes of *TaBSK3‐2B*.
**Table S7.** Accession numbers of the genes in this study.

## Data Availability

The RNA‐seq data that support the findings of this study are available in NCBI BioProject at https://www.ncbi.nlm.nih.gov/bioproject?db=bioproject, reference number PRJNA1394450. Other data are available in this paper and its Supporting Information files.

## References

[tpj71118-bib-0001] Bazhenov, M.S. , Divashuk, M.G. , Amagai, Y. , Watanabe, N. & Karlov, G.I. (2015) Isolation of the dwarfing *Rht‐B1p* (*Rht17*) gene from wheat and the development of an allele‐specific PCR marker. Molecular Breeding, 35, 213.

[tpj71118-bib-0002] Börner, A. , Plaschke, J. , Korzun, V. & Worland, A.J. (1996) The relationships between the dwarfing genes of wheat and rye. Euphytica, 89, 69–75.

[tpj71118-bib-0003] Borrill, P. , Mago, R. , Xu, T. , Ford, B. , Williams, S.J. , Derkx, A. et al. (2022) An autoactive NB‐LRR gene causes *Rht13* dwarfism in wheat. Proceedings of the National Academy of Sciences of the United States of America, 119, e2209875119. 10.1073/pnas.2209875119 PMC9860330 36417432

[tpj71118-bib-0004] Broman, K.W. , Wu, H. , Sen, Ś. & Churchill, G.A. (2003) R/QTL: QTL mapping in experimental crosses. Bioinformatics, 19, 889–890. 10.1093/bioinformatics/btg112 12724300

[tpj71118-bib-0005] Buss, W. , Ford, B.A. , Foo, E. , Schnippenkoetter, W. , Borrill, P. , Brooks, B. et al. (2020) Overgrowth mutants determine the causal role of gibberellin *GA2oxidaseA13* in *Rht12* dwarfism of wheat. Journal of Experimental Botany, 71, 7171–7178. 10.1093/jxb/eraa443 32949136

[tpj71118-bib-0006] Cadalen, T. , Sourdille, P. , Charmet, G. , Tixier, M.H. , Gay, G. , Boeuf, C. et al. (1998) Molecular markers linked to genes affecting plant height in wheat using a doubled‐haploid population. Theoretical and Applied Genetics, 96, 933–940.

[tpj71118-bib-0007] Chai, L. , Chen, Z. , Bian, R. , Zhai, H. , Cheng, X. , Peng, H. et al. (2018) Dissection of two quantitative trait loci with pleiotropic effects on plant height and spike length linked in coupling phase on the short arm of chromosome 2D of common wheat (*Triticum aestivum* L.). Theoretical and Applied Genetics, 131, 2621–2637. 10.1007/s00122-018-3177-4 30267114

[tpj71118-bib-0008] Chai, L. , Xin, M. , Dong, C. , Chen, Z. , Zhai, H. , Zhuang, J. et al. (2022) A natural variation in ribonuclease H‐like gene underlies *Rht8* to confer “green revolution” trait in wheat. Molecular Plant, 15, 377–380. 10.1016/j.molp.2022.01.013 35063659

[tpj71118-bib-0009] Chen, S. , Zhou, Y. , Chen, Y. & Gu, J. (2018) Fastp: an ultra‐fast all‐in‐one FASTQ preprocessor. Bioinformatics, 34, i884–i890. 10.1093/bioinformatics/bty560 PMC6129281 30423086

[tpj71118-bib-0010] Cheng, S. , Feng, C. , Wingen, L.U. , Cheng, H. , Riche, A.B. , Jiang, M. et al. (2024) Harnessing landrace diversity empowers wheat breeding. Nature, 632, 823–831. 10.1038/s41586-024-07682-9 PMC11338829 38885696

[tpj71118-bib-0011] Cui, C. , Lu, Q. , Zhao, Z. , Lu, S. , Duan, S. , Yang, Y. et al. (2022) The fine mapping of dwarf gene *Rht5* in bread wheat and its effects on plant height and main agronomic traits. Planta, 255, 114. 10.1007/s00425-022-03888-1 35507093

[tpj71118-bib-0097] Cui, X. , Gao, Y. , Guo, J. , Yu, T. , Zheng, W. , Liu, Y. et al. (2019). BES/BZR transcription factor TaBZR2 positively regulates drought responses by activation of TaGST1. Plant Physiology, 180, 605–620. 10.1104/pp.19.00100 PMC6501075 30842265

[tpj71118-bib-0012] Divashuk, M.G. , Vasilyev, A.V. , Bespalova, L.A. & Karlov, G.I. (2012) Identity of the *Rht‐11* and *Rht‐B1e* reduced plant height genes. Russian Journal of Genetics, 48, 761–763. 22988778

[tpj71118-bib-0013] Dong, H. , Li, D. , Yang, R. , Zhang, L. , Zhang, Y. , Liu, X. et al. (2023) GSK3 phosphorylates and regulates the green revolution protein Rht‐B1b to reduce plant height in wheat. The Plant Cell, 35, 1970–1983. 10.1093/plcell/koad090 PMC10226569 36945740

[tpj71118-bib-0014] Du, Y. , Chen, L. , Wang, Y. , Yang, Z. , Saeed, I. , Daoura, B.G. et al. (2018) The combination of dwarfing genes *Rht4* and *Rht8* reduced plant height, improved yield traits of rainfed bread wheat (*Triticum aestivum* L.). Field Crops Research, 215, 149–155.

[tpj71118-bib-0015] Duan, S. , Cui, C. , Chen, L. , Yang, Z. & Hu, Y. (2022) Fine mapping and candidate gene analysis of dwarf gene *Rht14* in durum wheat (*Triticum durum*). Functional & Integrative Genomics, 22, 141–152. 10.1007/s10142-021-00825-5 34981261

[tpj71118-bib-0016] Ehdaie, B. & Waines, J.G. (1996) Dwarfing genes, water‐use efficiency and agronomic performance of spring wheat. Canadian Journal of Plant Science, 76, 707–714.

[tpj71118-bib-0017] Ellis, M.H. , Rebetzke, G.J. , Azanza, F. , Richards, R.A. & Spielmeyer, W. (2005) Molecular mapping of gibberellin‐responsive dwarfing genes in bread wheat. Theoretical and Applied Genetics, 111, 423–430. 10.1007/s00122-005-2008-6 15968526

[tpj71118-bib-0018] Ellis, M.H. , Rebetzke, G.J. , Chandler, P. , Bonnett, D. , Spielmeyer, W. & Richards, R.A. (2004) The effect of different height reducing genes on the early growth of wheat. Functional Plant Biology, 31, 583–589. 10.1071/FP03207 32688930

[tpj71118-bib-0019] Fischer, R.A. & Maurer, R. (1978) Drought resistance in spring wheat cultivars. I. Grain yield responses. Australian Journal of Agricultural Research, 29, 897–912.

[tpj71118-bib-0020] Ford, B.A. , Foo, E. , Sharwood, R. , Karafiatova, M. , Vrána, J. , MacMillan, C. et al. (2018) *Rht18* semidwarfism in wheat is due to increased *GA2‐oxidaseA9* expression and reduced GA content. Plant Physiology, 177, 168–180. 10.1104/pp.18.00023 PMC5933146 29545269

[tpj71118-bib-0021] Fu, M. , Liu, S. , Che, Y. , Cui, D. , Deng, Z. , Li, Y. et al. (2024) Genome‐editing of a circadian clock gene *TaPRR95* facilitates wheat peduncle growth and heading date. Journal of Genetics and Genomics, 51, 1101–1110. 10.1016/j.jgg.2024.05.011 38849110

[tpj71118-bib-0022] Fujioka, S. , Noguchi, T. , Takatsuto, S. & Yoshida, S. (1998) Activity of brassinosteroids in the dwarf rice lamina inclination bioassay. Phytochemistry, 49, 1841–1848.

[tpj71118-bib-0023] Gasperini, D. , Greenland, A. , Hedden, P. , Dreos, R. , Harwood, W. & Griffiths, S. (2012) Genetic and physiological analysis of *Rht8* in bread wheat: an alternative source of semi‐dwarfism with a reduced sensitivity to brassinosteroids. Journal of Experimental Botany, 63, 4419–4436. 10.1093/jxb/ers138 PMC3421992 22791821

[tpj71118-bib-0024] Gill, M.S. , Phillips, A.L. , Tarkowská, D. , Addy, J. , Sokolowska, P. , Foulkes, M.J. et al. (2025) Induced variation in *BRASSINOSTEROID INSENSITIVE 1* (*BRI1*) confers a compact wheat architecture. BMC Plant Biology, 25, 700. 10.1186/s12870-025-06762-w PMC12105372 40419954

[tpj71118-bib-0025] Gooding, M.J. , Addisu, M. , Uppal, R.K. , Snape, J.W. & Jones, H.E. (2012) Effect of wheat dwarfing genes on nitrogen‐use efficiency. The Journal of Agricultural Science, 150, 3–22.

[tpj71118-bib-0026] Hedden, P. (2003) The genes of the green revolution. Trends in Genetics, 19, 5–9. 10.1016/s0168-9525(02)00009-4 12493241

[tpj71118-bib-0027] Hong, Z. , Ueguchi‐Tanaka, M. , Shimizu‐Sato, S. , Inukai, Y. , Fujioka, S. , Shimada, Y. et al. (2002) Loss‐of‐function of a rice brassinosteroid biosynthetic enzyme, C‐6 oxidase, prevents the organized arrangement and polar elongation of cells in the leaves and stem. The Plant Journal, 32, 495–508. 10.1046/j.1365-313x.2002.01438.x 12445121

[tpj71118-bib-0028] Hong, Z. , Ueguchi‐Tanaka, M. , Umemura, K. , Uozu, S. , Fujioka, S. , Takatsuto, S. et al. (2003) A rice brassinosteroid‐deficient mutant, *ebisu dwarf* (*d2*), is caused by a loss of function of a new member of cytochrome P450. The Plant Cell, 15, 2900–2910. 10.1105/tpc.014712 PMC282825 14615594

[tpj71118-bib-0029] Hoshikawa, K. (1989) Stem. In: Hoshikawa, K. (Ed.) The Growing Rice Plant. Tokyo: Nobunkyo, pp. 123–148.

[tpj71118-bib-0030] Ishida, Y. , Tsunashima, M. , Hiei, Y. & Komari, T. (2015) Wheat (*Triticum aestivum* L.) transformation using immature embryos. Methods in Molecular Biology, 1223, 189–198. 10.1007/978-1-4939-1695-5_15 25300841

[tpj71118-bib-0031] Jatayev, S. , Sukhikh, I. , Vavilova, V. , Smolenskaya, S.E. , Goncharov, N.P. , Kurishbayev, A. et al. (2020) Green revolution ‘stumbles’ in a dry environment: Dwarf wheat with *Rht* genes fails to produce higher grain yield than taller plants under drought. Plant, Cell & Environment, 43, 2355–2364. 10.1111/pce.13819 32515827

[tpj71118-bib-0032] Jiao, C. , Xie, X. , Hao, C. , Chen, L. , Xie, Y. , Garg, V. et al. (2025) Pan‐genome bridges wheat structural variations with habitat and breeding. Nature, 637, 384–393. 10.1038/s41586-024-08277-0 39604736

[tpj71118-bib-0033] Khalid, M.A. , Ali, Z. , Husnain, L.A. , Fiaz, S. , Saddique, M.A.B. , Merrium, S. et al. (2024) GA‐sensitive *Rht13* gene improves root architecture and osmotic stress tolerance in bread wheat. BMC Genomic Data, 25, 90. 10.1186/s12863-024-01272-4 PMC11515344 39449141

[tpj71118-bib-0034] Kim, D. , Paggi, J.M. , Park, C. , Bennett, C. & Salzberg, S.L. (2019) Graph‐based genome alignment and genotyping with HISAT2 and HISAT‐genotype. Nature Biotechnology, 37, 907–915. 10.1038/s41587-019-0201-4 PMC7605509 31375807

[tpj71118-bib-0035] Kim, T.W. , Guan, S. , Sun, Y. , Deng, Z. , Tang, W. , Shang, J. et al. (2009) Brassinosteroid signal transduction from cell‐surface receptor kinases to nuclear transcription factors. Nature Cell Biology, 11, 1254–1260. 10.1038/ncb1970 PMC2910619 19734888

[tpj71118-bib-0036] Konzak, C.F. (1976) A review of semidwarfing gene sources and a description of some new mutants useful for breeding short‐stature wheats. In: Bottino, P.J. (Ed.) Induced mutations in cross‐breeding. Vienna, Austria: International Atomic Energy Agency, pp. 79–93.

[tpj71118-bib-0037] Kumar, A. , Sandhu, N. , Kumar, P. , Pruthi, G. , Singh, J. , Kaur, S. et al. (2022) Genome‐wide identification and in silico analysis of *NPF*, *NRT2*, *CLC* and *SLAC1*/*SLAH* nitrate transporters in hexaploid wheat (*Triticum aestivum*). Scientific Reports, 12, 11227. 10.1038/s41598-022-15202-w PMC9250930 35781289

[tpj71118-bib-0038] Li, H. & Durbin, R. (2009) Fast and accurate short read alignment with burrows‐wheeler transform. Bioinformatics, 25, 1754–1760. 10.1093/bioinformatics/btp324 PMC2705234 19451168

[tpj71118-bib-0039] Li, Q. , Wang, C. , Jiang, L. , Shuo, L. , Sun, S.S.M. & He, J. (2012) An interaction between BZR1 and DELLAs mediates direct signaling crosstalk between brassinosteroids and gibberellins in *Arabidopsis* . Science Signaling, 5, ra72. 10.1126/scisignal.2002908 23033541

[tpj71118-bib-0040] Li, R. , Liu, J. , Chai, L. , Du, D. , Yang, W. , Zhu, J. et al. (2025) Natural variation in *TaERF‐A1* confers semi‐dwarf and lodging‐resistant plant architecture in wheat. Plant Communications, 6, 101194. 10.1016/j.xplc.2024.101194 PMC11956107 39563037

[tpj71118-bib-0041] Li, X. , Lan, S. , Liu, Y. , Gale, M. & Worland, T. (2006) Effects of different *Rht‐B1b*, *Rht‐D1b* and *Rht‐B1c* and dwarfing genes on agronomic characteristics in wheat. Cereal Research Communications, 34, 919–924.

[tpj71118-bib-0042] Liu, H. , Zhi, L. , Qiao, H. , Niu, Y. , Shang, J. , Zhang, H. et al. (2025) *PEDUNCLE LENGTH 1* regulates wheat peduncle elongation through brassinosteroid signaling pathway. Molecular Plant, 18, 1606–1609. 10.1016/j.molp.2025.09.003 40913312

[tpj71118-bib-0043] Liu, X. , Lan, J. , Huang, Y. , Cao, P. , Zhou, C. , Ren, Y. et al. (2018) WSL5, a pentatricopeptide repeat protein, is essential for chloroplast biogenesis in rice under cold stress. Journal of Experimental Botany, 69, 3949–3961. 10.1093/jxb/ery214 PMC6054151 29893948

[tpj71118-bib-0044] Liu, Y. , Zhang, J. , Hu, Y. & Chen, J. (2017) Dwarfing genes *Rht4* and *Rht‐B1b* affect plant height and key agronomic traits in common wheat under two water regimes. Field Crops Research, 204, 242–248.

[tpj71118-bib-0045] Livak, K.K. & Schmittgen, T.D. (2001) Analysis of relative gene expression data using real‐time quantitative PCR and the 2^−ΔΔCT^ method. Methods, 25, 402–408. 10.1006/meth.2001.1262 11846609

[tpj71118-bib-0046] Love, M.I. , Huber, W. & Anders, S. (2014) Moderated estimation of fold change and dispersion for RNA‐seq data with DESeq2. Genome Biology, 15, 550. 10.1186/s13059-014-0550-8 PMC4302049 25516281

[tpj71118-bib-0047] Lu, Q. , Lu, S. , Wang, M. , Cui, C. , Condon, A.G. , Jatayev, S. et al. (2022) The exogenous GA_3_ greatly affected the grain‐filling process of semi‐dwarf gene *Rht4* in bread wheat. Physiologia Plantarum, 174, e13725. 10.1111/ppl.13725 35642076

[tpj71118-bib-0048] Luo, C. , Wang, L. , Wu, Y. , Cai, J. , Peng, B. , Chen, J. et al. (2026) One primer pair for all: a standardized vector toolbox for protein–protein interactions and protein localization in plants. Plant, Cell & Environment, 49, 352–365. 10.1111/pce.70219 41031466

[tpj71118-bib-0049] Lyu, J. , Wang, D. , Sun, N. , Yang, F. , Li, X. , Mu, J. et al. (2024) The TaSnRK1‐TabHLH489 module integrates brassinosteroid and sugar signalling to regulate the grain length in bread wheat. Plant Biotechnology Journal, 22, 1989–2006. 10.1111/pbi.14319 PMC11182588 38412139

[tpj71118-bib-0050] McKenna, A. , Hanna, M. , Banks, E. , Sivachenko, A. , Cibulskis, K. , Kernytsky, A. et al. (2010) The genome analysis toolkit: a MapReduce framework for analyzing next‐generation DNA sequencing data. Genome Research, 20, 1297–1303. 10.1101/gr.107524.110 PMC2928508 20644199

[tpj71118-bib-0051] Miao, L. , Wang, X. , Yu, C. , Ye, C. , Yan, Y. & Wang, H.S. (2024) What factors control plant height? Journal of Integrative Agriculture, 23, 1803–1824.

[tpj71118-bib-0052] Monneveux, P. , Jing, R. & Misra, S.C. (2012) Phenotyping for drought adaptation in wheat using physiological traits. Frontiers in Physiology, 3, 429. 10.3389/fphys.2012.00429 PMC3499878 23181021

[tpj71118-bib-0053] Niu, M. , Wang, H. , Yin, W. , Meng, W. , Xiao, Y. , Liu, D. et al. (2022) Rice DWARF AND LOW‐TILLERING and the homeodomain protein OSH15 interact to regulate internode elongation via orchestrating brassinosteroid signaling and metabolism. The Plant Cell, 34, 3754–3772. 10.1093/plcell/koac196 PMC9516196 35789396

[tpj71118-bib-0054] Nolan, T.M. , Vukasinovic, N. , Liu, D.R. , Russinova, E. & Yin, Y.H. (2020) Brassinosteroids: multidimensional regulators of plant growth, development, and stress responses. The Plant Cell, 32, 295–318. 10.1105/tpc.19.00335 PMC7008487 31776234

[tpj71118-bib-0055] Olszewski, N. , Sun, T.P. & Gubler, F. (2002) Gibberellin signaling: biosynthesis, catabolism, and response pathways. The Plant Cell, 14, S61–S80. 10.1105/tpc.010476 PMC151248 12045270

[tpj71118-bib-0056] Peng, J.R. , Richards, D.E. , Hartley, N.M. , Murphy, G.P. , Devos, K.M. , Flintham, J.E. et al. (1999) ‘Green revolution’ genes encode mutant gibberellin response modulators. Nature, 400, 256–261. 10.1038/22307 10421366

[tpj71118-bib-0057] Qin, Z. , Lv, H. , Zhu, X. , Meng, C. , Quan, T. , Wang, M. et al. (2013) Ectopic expression of a wheat WRKY transcription factor gene *TaWARKY71‐1* results in hyponastic leaves in *Arabidopsis thaliana* . PLoS One, 8, e63033. 10.1371/journal.pone.0063033 PMC3650005 23671653

[tpj71118-bib-0058] Rebetzke, G.J. , Richards, R.A. , Fettell, N.A. , Long, M. , Condon, A.G. , Forrester, R.I. et al. (2007) Genotypic increases in coleoptile length improves stand establishment, vigour and grain yield of deep‐sown wheat. Field Crops Research, 100, 10–23.

[tpj71118-bib-0059] Rebetzke, G.J. , Richards, R.A. , Fischer, V.M. & Mickelson, B.J. (1999) Breeding long coleoptile, reduced height wheats. Euphytica, 106, 159–168.

[tpj71118-bib-0060] Ren, H. , Willige, B.C. , Jaillais, Y. , Geng, S. , Park, M.Y. , Gray, W.M. et al. (2019) BRASSINOSTEROID‐SIGNALING KINASE 3, a plasma membrane‐associated scaffold protein involved in early brassinosteroid signaling. PLoS Genetics, 15, e1007904. 10.1371/journal.pgen.1007904 PMC6336344 30615605

[tpj71118-bib-0061] Robil, J.M. , Awale, P. , McSteen, P. & Best, N.B. (2025) Gibberellins: extending the green revolution. Journal of Experimental Botany, 76, 1837–1853. 10.1093/jxb/erae476 PMC12066124 39570614

[tpj71118-bib-0062] Robinson, J.T. , Thorvaldsdottir, H. , Winckler, W. , Guttman, M. , Lander, E.S. , Getz, G. et al. (2011) Integrative genomics viewer. Nature Biotechnology, 29, 24–26. 10.1038/nbt.1754 PMC3346182 21221095

[tpj71118-bib-0063] Sasaki, A. , Ashikari, M. , Ueguchi‐Tanaka, M. , Itoh, H. , Nishimura, A. , Swapan, D. et al. (2002) Green revolution: a mutant gibberellin‐synthesis gene in rice. Nature, 416, 701–702. 10.1038/416701a 11961544

[tpj71118-bib-0064] Song, L. , Liu, J. , Cao, B. , Liu, B. , Zhang, X. , Chen, Z. et al. (2023) Reducing brassinosteroid signalling enhances grain yield in semi‐dwarf wheat. Nature, 617, 118–124. 10.1038/s41586-023-06023-6 PMC10156601 37100915

[tpj71118-bib-0065] Sparkes, I.A. , Runions, J. , Kearns, A. & Hawes, C. (2006) Rapid, transient expression of fluorescent fusion proteins in tobacco plants and generation of stably transformed plants. Nature Protocols, 1, 2019–2025. 10.1038/nprot.2006.286 17487191

[tpj71118-bib-0066] Srinivasachary , Gosman, N. , Steed, A. , Hollins, T.W. , Bayles, R. , Jennings, P. et al. (2009) Semi‐dwarfing *Rht‐B1* and *Rht‐D1* loci of wheat differ significantly in their influence on resistance to Fusarium head blight. Theoretical and Applied Genetics, 118, 695–702. 10.1007/s00122-008-0930-0 19034409

[tpj71118-bib-0067] Sun, L. , Yang, W. , Li, Y. , Shan, Q. , Ye, X. , Wang, D. et al. (2019) A wheat dominant dwarfing line with *Rht12*, which reduces stem cell length and affects gibberellic acid synthesis, is a 5AL terminal deletion line. The Plant Journal, 97, 887–900. 10.1111/tpj.14168 30466195

[tpj71118-bib-0068] Sun, Y. , Fan, X. , Cao, D. , Tang, W. , He, K. , Zhu, J. et al. (2010) Integration of brassinosteroid signal transduction with the transcription network for plant growth regulation in *Arabidopsis* . Developmental Cell, 19, 765–777. 10.1016/j.devcel.2010.10.010 PMC3018842 21074725

[tpj71118-bib-0069] Tanabe, S. , Ashikari, M. , Fujioka, S. , Takatsuto, S. , Yoshida, S. , Yano, M. et al. (2005) A novel cytochrome P450 is implicated in brassinosteroid biosynthesis via the characterization of a rice dwarf mutant, *dwarf11*, with reduced seed length. The Plant Cell, 17, 776–790. 10.1105/tpc.104.024950 PMC1069698 15705958

[tpj71118-bib-0070] Tang, W. , Kim, T.W. , Oses‐Prieto, J.A. , Sun, Y. , Deng, Z.P. , Zhu, S.W. et al. (2008) BSKs mediate signal transduction from the receptor kinase BRI1 in *Arabidopsis* . Science, 321, 557–560. 10.1126/science.1156973 PMC2730546 18653891

[tpj71118-bib-0071] Tian, P. , Liu, J. , Yan, B. , Li, S. , Lei, B. , Shen, R. et al. (2022) OsBSK3 positively regulates grain length and weight by inhibiting the phosphatase activity of OsPPKL1. Plants, 11, 1586. 10.3390/plants11121586 PMC9229280 35736737

[tpj71118-bib-0072] Tian, X. , Wen, W. , Xie, L. , Fu, L. , Xu, D. , Fu, C. et al. (2017) Molecular mapping of reduced plant height gene *Rht24* in bread wheat. Frontiers in Plant Science, 8, 1379. 10.3389/fpls.2017.01379 PMC5550838 28848582

[tpj71118-bib-0073] Tian, X. , Xia, X. , Xu, D. , Liu, Y. , Xie, L. , Hassan, M.A. et al. (2022) *Rht24b*, an ancient variation of *TaGA2ox‐A9*, reduces plant height without yield penalty in wheat. New Phytologist, 233, 738–750. 10.1111/nph.17808 34655489

[tpj71118-bib-0074] Tong, H. , Jin, Y. , Liu, W. , Li, F. , Fang, J. , Yin, Y. et al. (2009) DWARF AND LOW‐TILLERING, a new member of the GRAS family, plays positive roles in brassinosteroid signaling in rice. The Plant Journal, 58, 803–816. 10.1111/j.1365-313X.2009.03825.x 19220793

[tpj71118-bib-0075] Tong, H. , Xiao, Y. , Liu, D. , Gao, S. , Liu, L. , Yin, Y. et al. (2014) Brassinosteroid regulates cell elongation by modulating gibberellin metabolism in rice. The Plant Cell, 26, 4376–4393. 10.1105/tpc.114.132092 PMC4277228 25371548

[tpj71118-bib-0076] Uppal, R.K. & Gooding, M.J. (2013) Gibberellin‐responsive and ‐insensitive dwarfing alleles on wheat performance in contrasting tillage systems. Field Crops Research, 141, 55–62.

[tpj71118-bib-0077] van de Velde, K. , Thomas, S.G. , Heyse, F. , Kaspar, R. , van der Straeten, D. & Rohde, A. (2021) N‐terminal truncated RHT‐1 proteins generated by translational reinitiation cause semi‐dwarfing of wheat green revolution alleles. Molecular Plant, 14, 679–687. 10.1016/j.molp.2021.01.002 33422695

[tpj71118-bib-0078] Vikhe, P. , Venkatesan, S. , Chavan, A. , Tamhankar, S. & Patil, R. (2019) Mapping of dwarfing gene *Rht14* in durum wheat and its effect on seedling vigor, internode length and plant height. The Crop Journal, 7, 187–197.

[tpj71118-bib-0079] Wang, D. , Li, Y. , Wang, H. , Xu, Y. , Yang, Y. , Zhou, Y. et al. (2023) Boosting wheat functional genomics via an indexed EMS mutant library of KN9204. Plant Communications, 4, 100593. 10.1016/j.xplc.2023.100593 PMC10363553 36945776

[tpj71118-bib-0080] Wang, J. , Liu, Q. & Li, Q. (2025) DELLA family proteins function beyond the GA pathway. Trends in Plant Science, 30, 352–355. 10.1016/j.tplants.2025.01.007 39966013

[tpj71118-bib-0081] Wang, X. & Chory, J. (2006) Brassinosteroids regulate dissociation of BKI1, a negative regulator of BRI1 signaling, from the plasma membrane. Science, 313, 1118–1122. 10.1126/science.1127593 16857903

[tpj71118-bib-0082] Wang, Z. , Bai, M. , Oh, E. & Zhu, J. (2012) Brassinosteroid signaling network and regulation of photomorphogenesis. Annual Review of Genetics, 46, 701–724. 10.1146/annurev-genet-102209-163450 23020777

[tpj71118-bib-0083] Xiong, H. , Zhou, C. , Fu, M. , Guo, H. , Xie, Y. , Zhao, L. et al. (2022) Cloning and functional characterization of *Rht8*, a “green revolution” replacement gene in wheat. Molecular Plant, 15, 373–376. 10.1016/j.molp.2022.01.014 35063661

[tpj71118-bib-0084] Yamaguchi, S. (2008) Gibberellin metabolism and its regulation. Annual Review of Plant Biology, 59, 225–251. 10.1146/annurev.arplant.59.032607.092804 18173378

[tpj71118-bib-0085] Yamamuro, C. , Ihara, Y. , Wu, X. , Noguchi, T. , Fujioka, S. , Takatsuto, S. et al. (2000) Loss of function of a rice brassinosteroid insensitive1 homolog prevents internode elongation and bending of the lamina joint. The Plant Cell, 12, 1591–1605. 10.1105/tpc.12.9.1591 PMC149072 11006334

[tpj71118-bib-0086] Yin, W. , Dong, N. , Li, X. , Yang, Y. , Lu, Z. , Zhou, W. et al. (2025) Understanding brassinosteroid‐centric phytohormone interactions for crop improvement. Journal of Integrative Plant Biology, 67, 563–581. 10.1111/jipb.13849 39927447

[tpj71118-bib-0087] Yoo, S.D. , Cho, Y.H. & Sheen, J. (2007) Mesophyll protoplasts: a versatile cell system for transient gene expression analysis. Nature Protocols, 2, 1565–1572. 10.1038/nprot.2007.199 17585298

[tpj71118-bib-0088] Zadoks, J.C. , Chang, T.T. & Konzak, C.F. (1974) A decimal code for growth stages of cereals. Weed Research, 14, 415–421.

[tpj71118-bib-0089] Zhang, B. , Wang, X. , Zhao, Z. , Wang, R. , Huang, X. , Zhu, Y. et al. (2016) OsBRI1 activates BR signaling by preventing binding between the TPR and kinase domains of OsBSK3 via phosphorylation. Plant Physiology, 170, 1149–1161. 10.1104/pp.15.01668 PMC4734578 26697897

[tpj71118-bib-0090] Zhang, J. , Li, C. , Zhang, W. , Zhang, X. , Mo, Y. , Tranquilli, G.E. et al. (2023) Wheat plant height locus *RHT25* encodes a PLATZ transcription factor that interacts with DELLA (RHT1). Proceedings of the National Academy of Sciences of the United States of America, 120, e2300203120. 10.1073/pnas.2300203120 PMC10175819 37126674

[tpj71118-bib-0091] Zhang, S. , Zhang, R. , Song, G. , Gao, J. , Li, W. , Han, X. et al. (2018) Targeted mutagenesis using the *Agrobacterium tumefaciens*‐mediated CRISPR‐Cas9 system in common wheat. BMC Plant Biology, 18, 302. 10.1186/s12870-018-1496-x PMC6258442 30477421

[tpj71118-bib-0092] Zhang, Y. , Li, J. , Zhao, M. , Gong, Z. , Hu, W. , Wang, J. et al. (2025) Characterization of the *brittle culm 1* in hexaploid wheat reveals potential for enhanced biomass utilization. Industrial Crops and Products, 237, 122275.

[tpj71118-bib-0093] Zhao, K. , Xiao, J. , Liu, Y. , Chen, S. , Yuan, C. , Cao, A. et al. (2018) Rht23 (5Dq') likely encodes a Q homeologue with pleiotropic effects on plant height and spike compactness. Theoretical and Applied Genetics, 131, 1825–1834. 10.1007/s00122-018-3115-5 29855673

[tpj71118-bib-0094] Zhao, Z. , Wang, E. , Kirkegaard, J.A. & Rebetzke, G.J. (2022) Novel wheat varieties facilitate deep sowing to beat the heat of changing climates. Nature Climate Change, 12, 291–296.

[tpj71118-bib-0095] Zhu, T. , Wang, L. , Rimbert, H. , Rodriguez, J.C. , Deal, K.R. , de Oliveira, R. et al. (2021) Optical maps refine the bread wheat *Triticum aestivum* cv. Chinese Spring genome assembly. The Plant Journal, 107, 303–314. 10.1111/tpj.15289 PMC8360199 33893684

