# Supplementary material for: Deletion of TaBSK3‐2B attenuates BR signaling to regulate the Rht4 semi‐dwarf phenotype and drought tolerance in wheat
Source: Plant J. 2026 Sep 17;127(5):e71118. doi: 10.1111/tpj.71118 (PMC13584235; doi:10.1111/tpj.71118)
Supplement: Supplementary file 1 [file TPJ-127-0-s001.pdf]

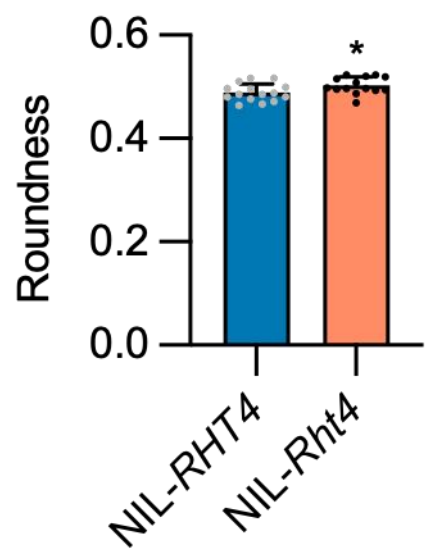

Figure S1 Grain roundness of NIL-*RHT4* and NIL-*Rht4*.

Quantification of grain roundness between NIL-*RHT4* and NIL-*Rht4*. Data are presented as means ± SD, $n = 15$. *$P < 0.05$, Student's *t*-test.

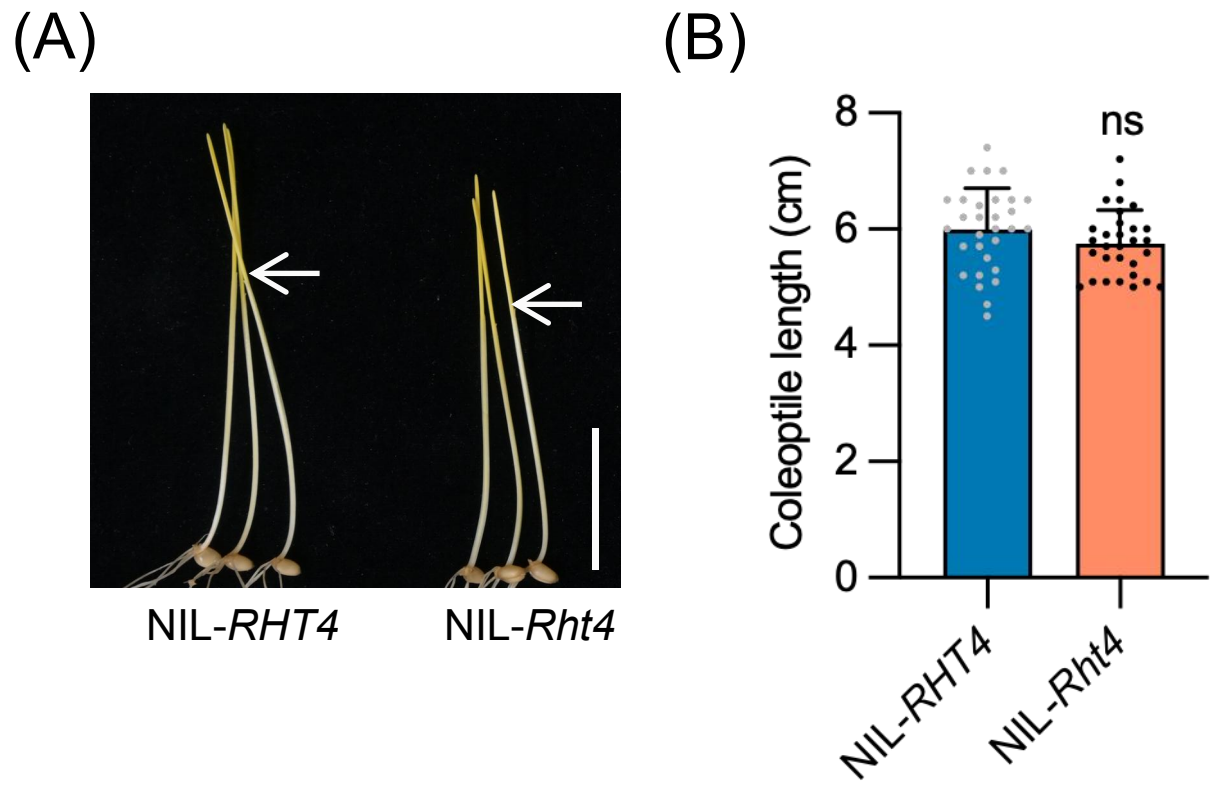

Figure S2 Coleoptile length of NIL-*RHT4* and NIL-*Rht4*.
(A) Coleoptile phenotypes of NIL-*RHT4* and NIL-*Rht4* seedlings grown in darkness for 7 days. Arrows indicate the coleoptile tips. Bar = 3 cm. (B) Statistical analysis of the coleoptile length between NIL-*RHT4* and NIL-*Rht4*. Data are presented as means ± SD, n = 30, ns, not significant by Student's t-test ($P < 0.05$).

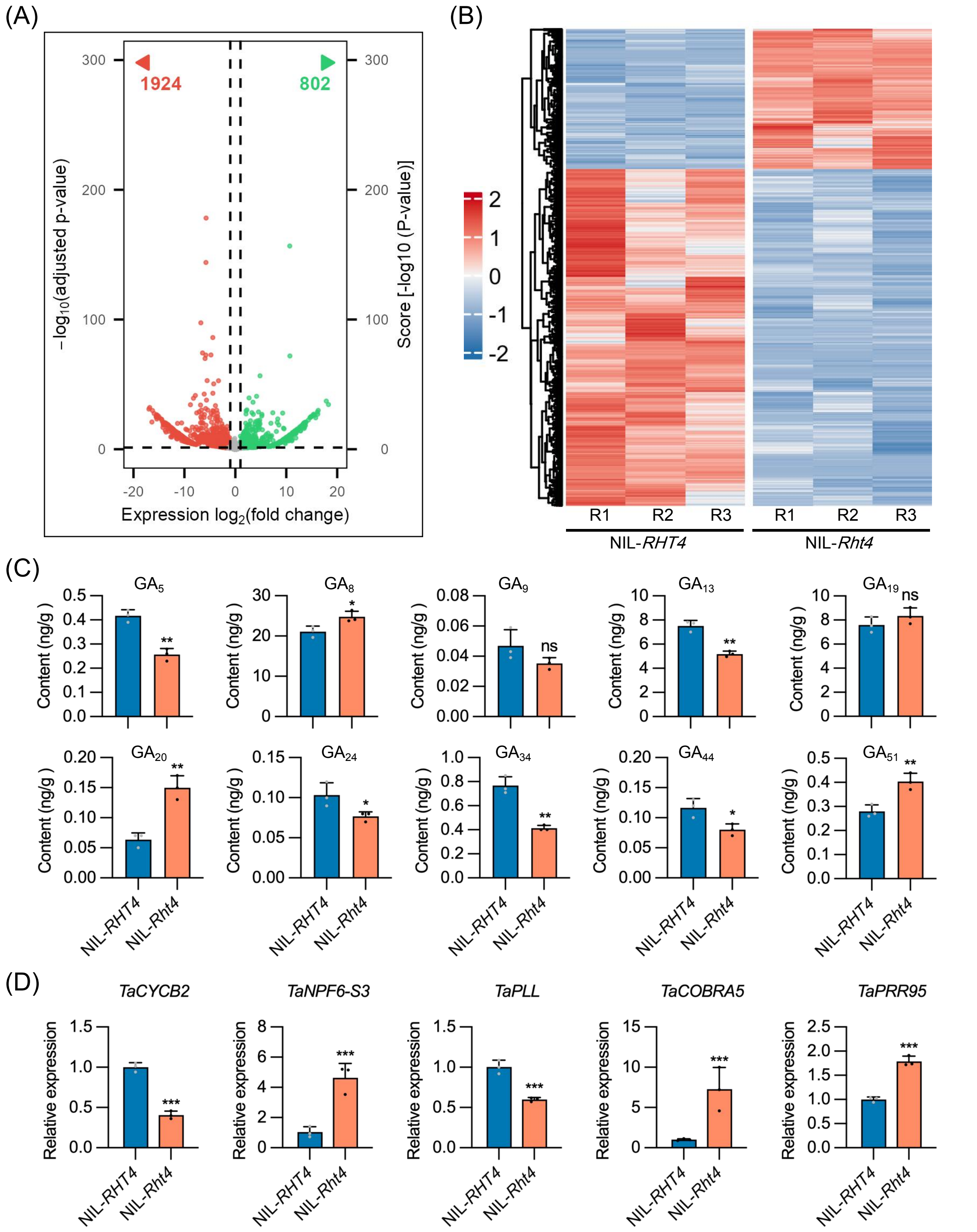

Figure S3 Transcriptome analysis and gibberellins (GAs) content measurement of NIL-*RHT4* and NIL-*Rht4* lines.

(A) Volcano plot showing 1924 down-regulated differentially expressed genes (DEGs) and 802 up-regulated DEGs identified between NIL-*RHT4* and NIL-*Rht4*. (B) Heatmap of the DEGs among three replicates between NIL-*RHT4* and NIL-*Rht4*. (C) Measurement of endogenous GA isoforms in peduncles from NIL-*RHT4* and NIL-*Rht4* at heading stage. (D) RT-qPCR revealed the transcriptional changes of cell growth-related genes in the peduncles of NIL-*RHT4* and NIL-*Rht4*. Data in (C)-(D) are presented as the means ± SD, $n = 3$. *$P < 0.05$, **$P < 0.01$, ***$P < 0.001$, Student's *t*-test.

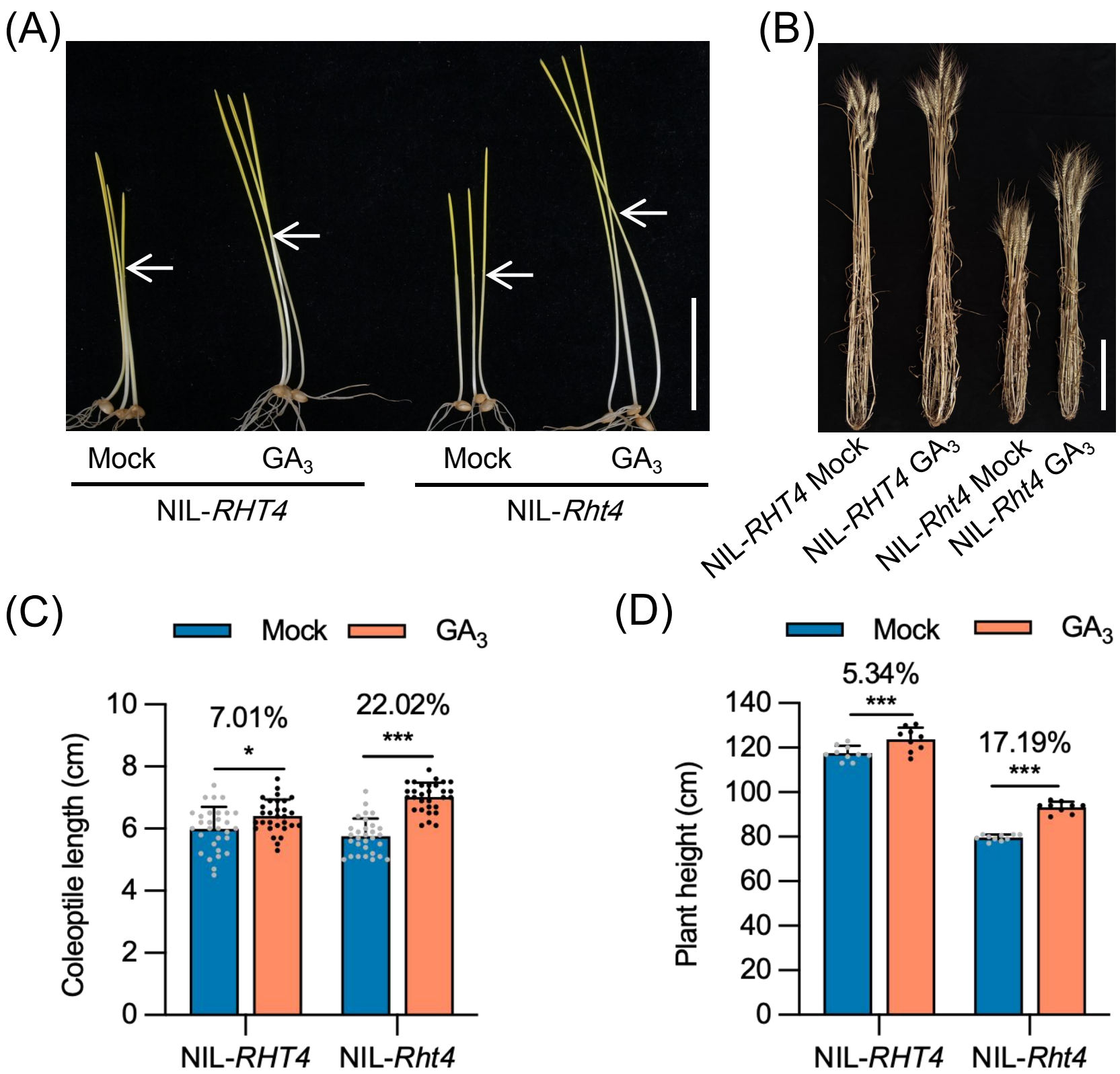

Figure S4 Response of NIL-*RHT4* and NIL-*Rht4* to exogenous GA_3 treatment.
(A) Coleoptile length of NIL-*Rht4* and NIL-*RHT4* upon exogenous GA_3 treatment. Bar = 3 cm. (B) Plant height at the mature stage of NIL-*RHT4* and NIL-*Rht4* upon exogenous GA_3 treatment. Bar = 10 cm. (C-D) Statistical analysis of the coleoptile length and plant height between NIL-*Rht4* and NIL-*RHT4* after applying with GA_3. Data in (C)-(D) are presented as the means ± SD, n = 30 in (C) and n = 10 in (D). *$P < 0.05$, **$P < 0.01$, ***$P < 0.001$, Student's t-test.

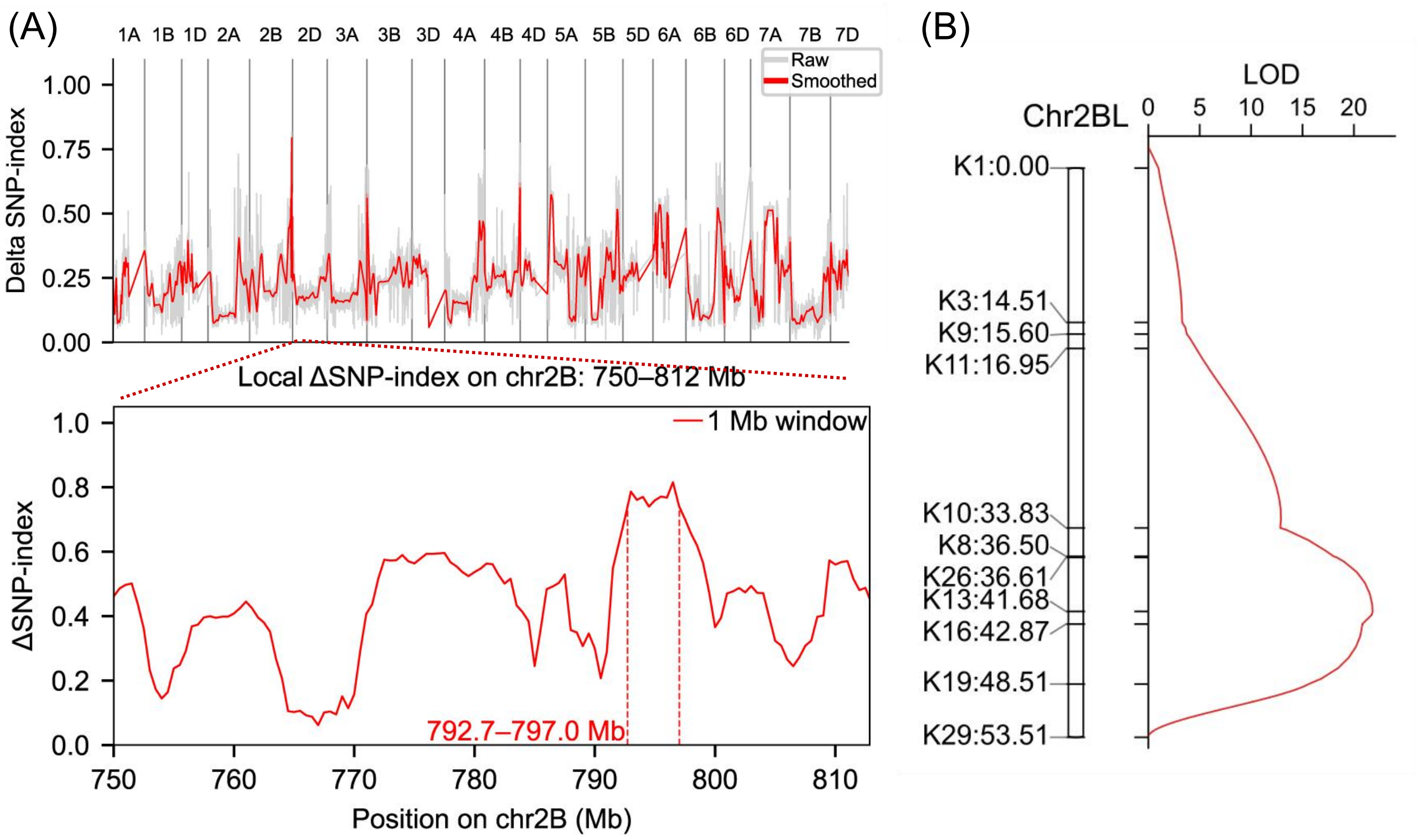

Figure S5 Preliminary mapping of *Rht4* using bulked-segregant analysis sequencing (BSA-seq) and quantitative trait loci (QTL) mapping.

(A) Genome-wide ΔSNP-index values calculated from whole-genome resequencing-based BSA-seq. A prominent peak was detected between 792.7 and 797 Mb on chromosome 2B. (B) A linkage map of chromosome 2B (bottom panel) and the LOD score profile from QTL analysis of plant height in the F_2 population (top panel).

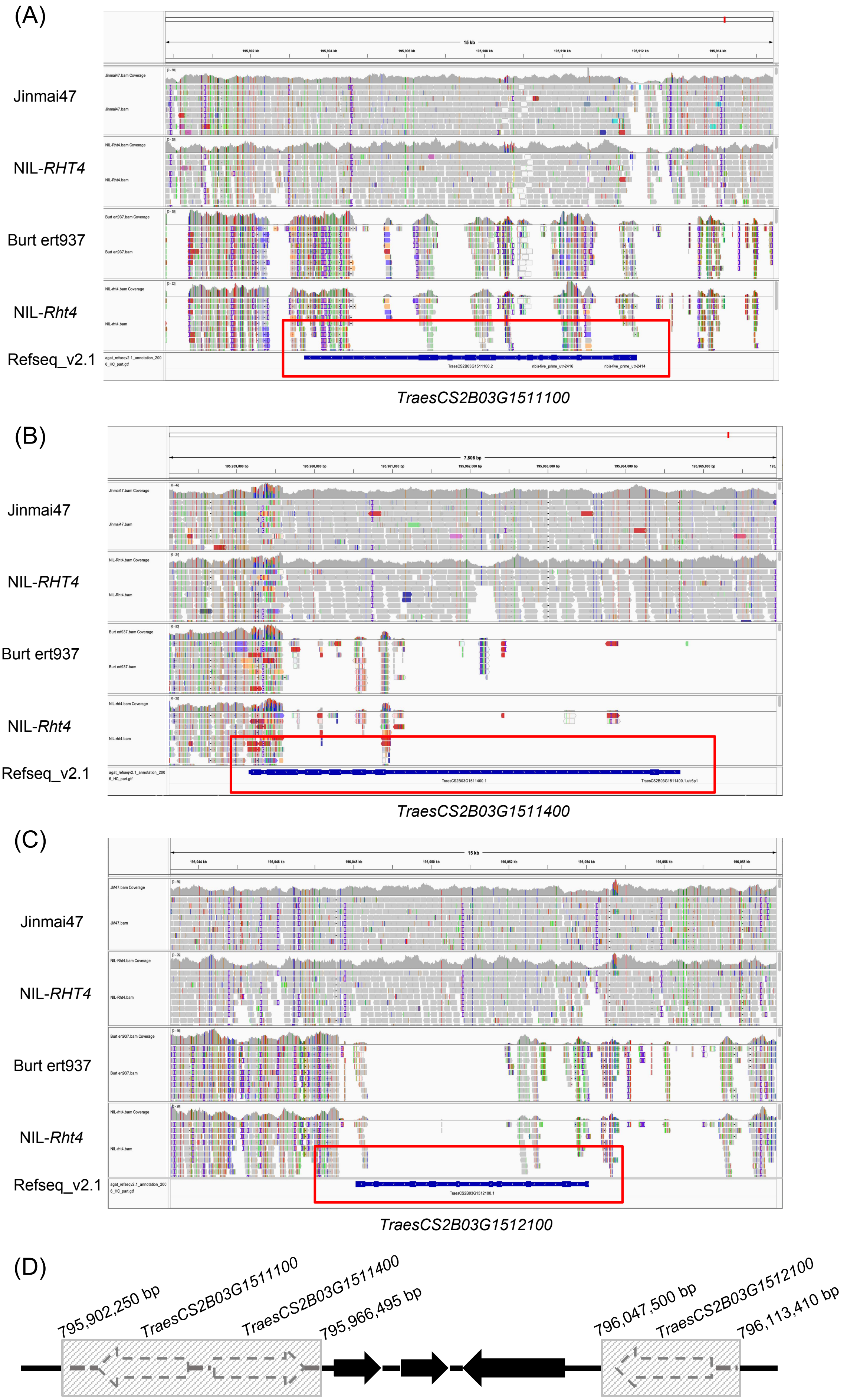

Figure S6 Whole-genome resequencing revealed gene deletions in the dwarf NIL-*Rht4* line and parental Burt ert937 in comparison with NIL-*RHT4* and Jinmai47.
(A-C) Sequence alignments showed that the *TraesCS2B03G1511100* (A), *TraesCS2B03G1511400* (B) and *TraesCS2B03G1512100* (C) were deleted (high-lighted by red boxes) in the candidate region of *Rht4* locus in Burt ert937 and NIL-*Rht4*. (D) Schematic representation of the gene deletions at *Rht4* candidate interval. Two large-fragment deletions are highlighted with dashed boxes, and dashed arrows indicate deleted genes.

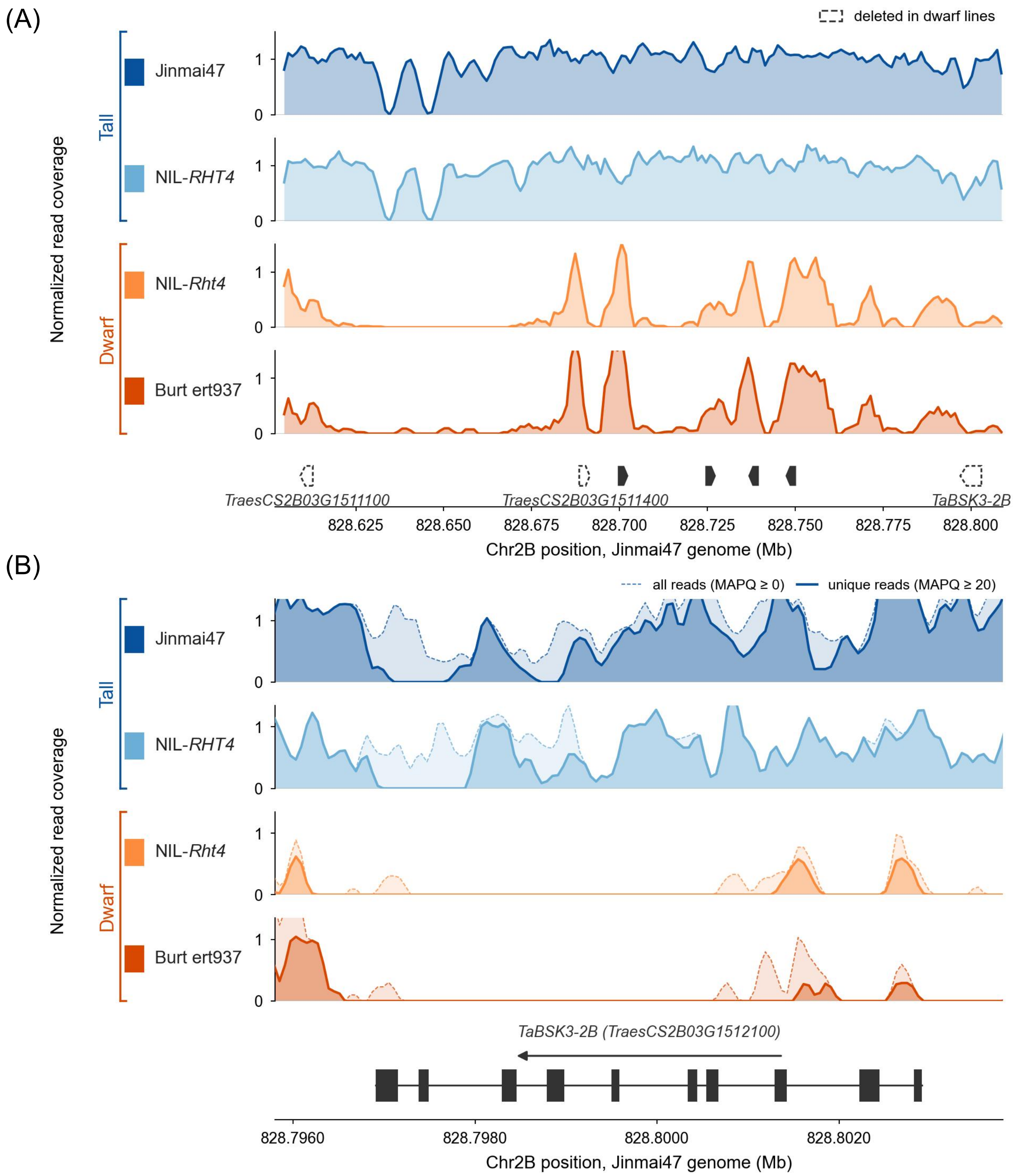

Figure S7 Whole-genome resequencing read-depth analysis of the *Rht4* candidate region. (A) Uniquely-mapped (MAPQ ≥ 20) coverage across the candidate region (828.61~828.81 Mb, Jinmai47 assembly), which contains *TraesCS2B03G1511100*, *TraesCS2B03G1511400* and *TraesCS2B03G1512100* (*TaBSK3-2B*). Dashed arrows indicated genes deleted in the Burt ert937 and NIL-*Rht4* revealed by whole-genome resequencing. (B) Resequencing read-depth analysis of the *TaBSK3-2B* locus. Dashed lines represent all mapped reads (MAPQ ≥ 0), whereas solid lines and shaded areas represent uniquely mapped reads (MAPQ ≥ 20). Black rectangles represent exons and black lines represent introns.

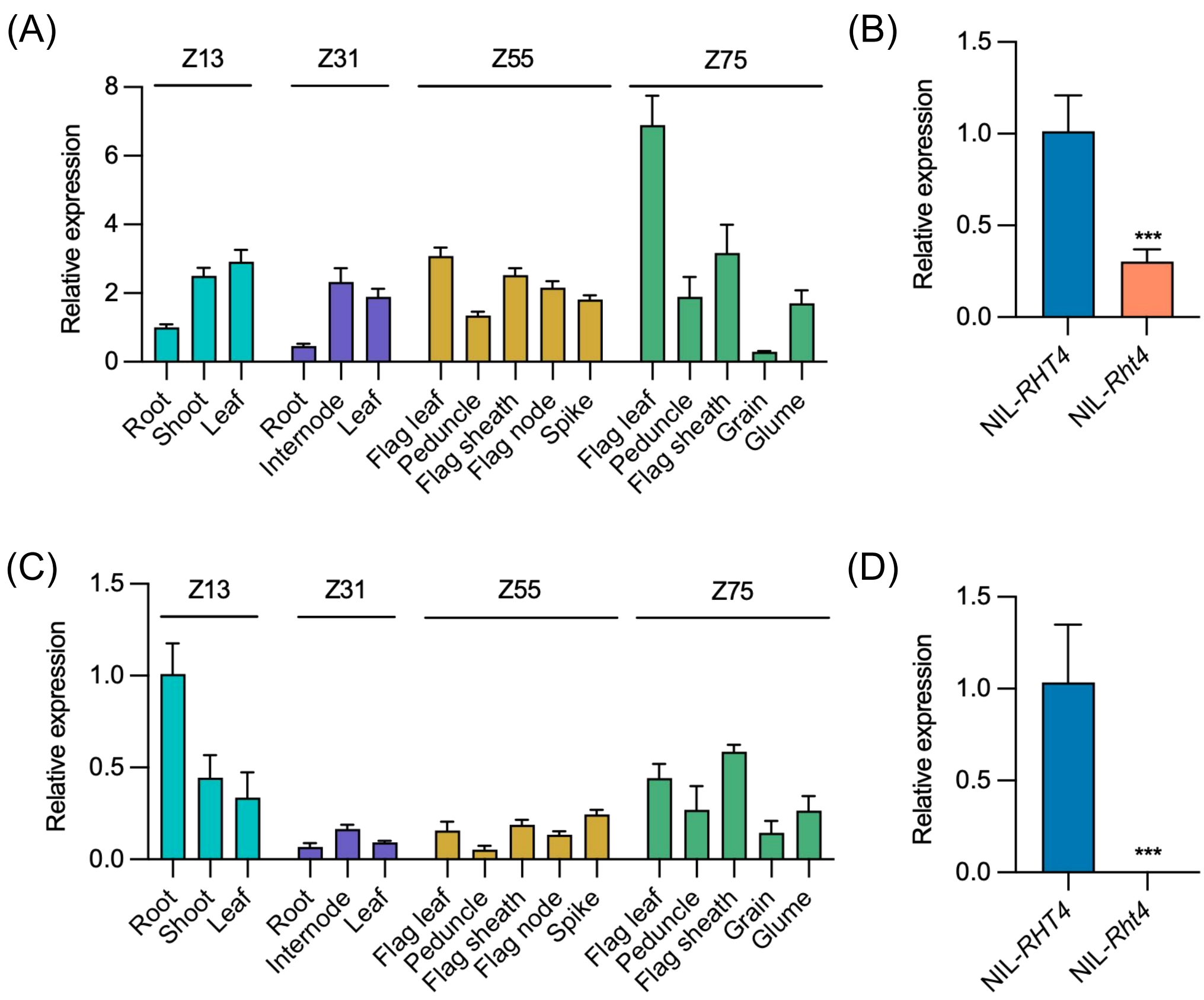

Figure S8 Expression patterns of *TraesCS2B03G1511100* and *TraesCS2B03G1511400.* (A, B) Relative expression of *TraesCS2B03G1511100* in various tissues and organs in Chinese spring (A) and peduncles of NIL-*RHT4* and NIL-*Rht4* (B). (C, D) Relative expression of *TraesCS2B03G1511400* in various tissues and organs in Chinese spring wheat (C) and peduncles of NIL-*RHT4* and NIL-*Rht4* (D). Data are presented as means ± SD, *n* = 3. Z13, three-leaf stage. Z31, jointing stage. Z55, heading stage. Z75, grain filling stage.

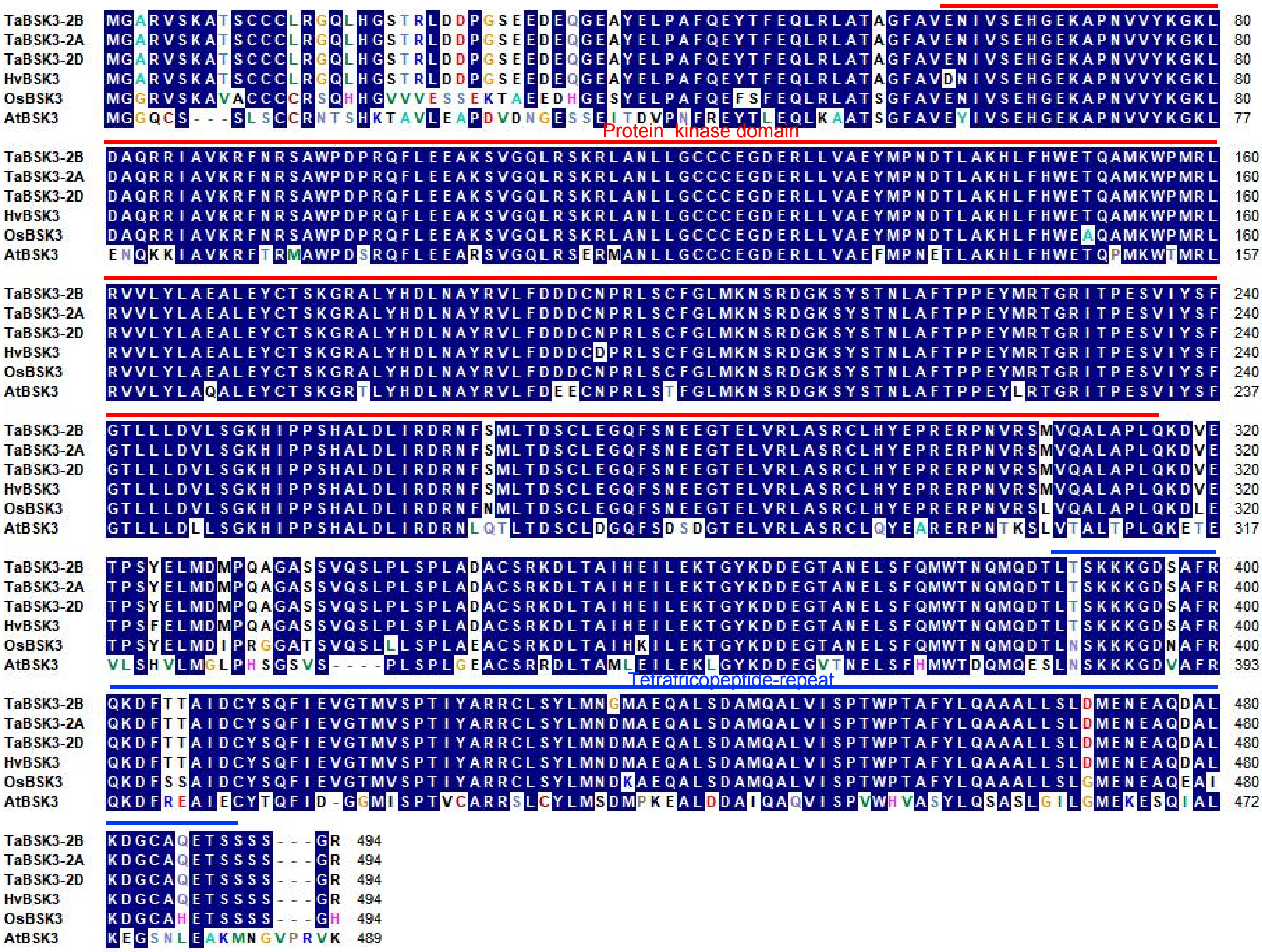

Figure S9 Multiple sequence alignment of BSK3 and its homologs across plant species.
Protein sequence alignment of TaBSK3-2B and its homologs in *Triticum aestivum* (Ta), *Hordeum vulgare* (Hv), *Arabidopsis thaliana* (At) and *Oryza sativa* (Os). The kinase domain and conserved tetratricopeptide-repeat (TPR) motif of BSK proteins were highlighted with red and blue, respectively.

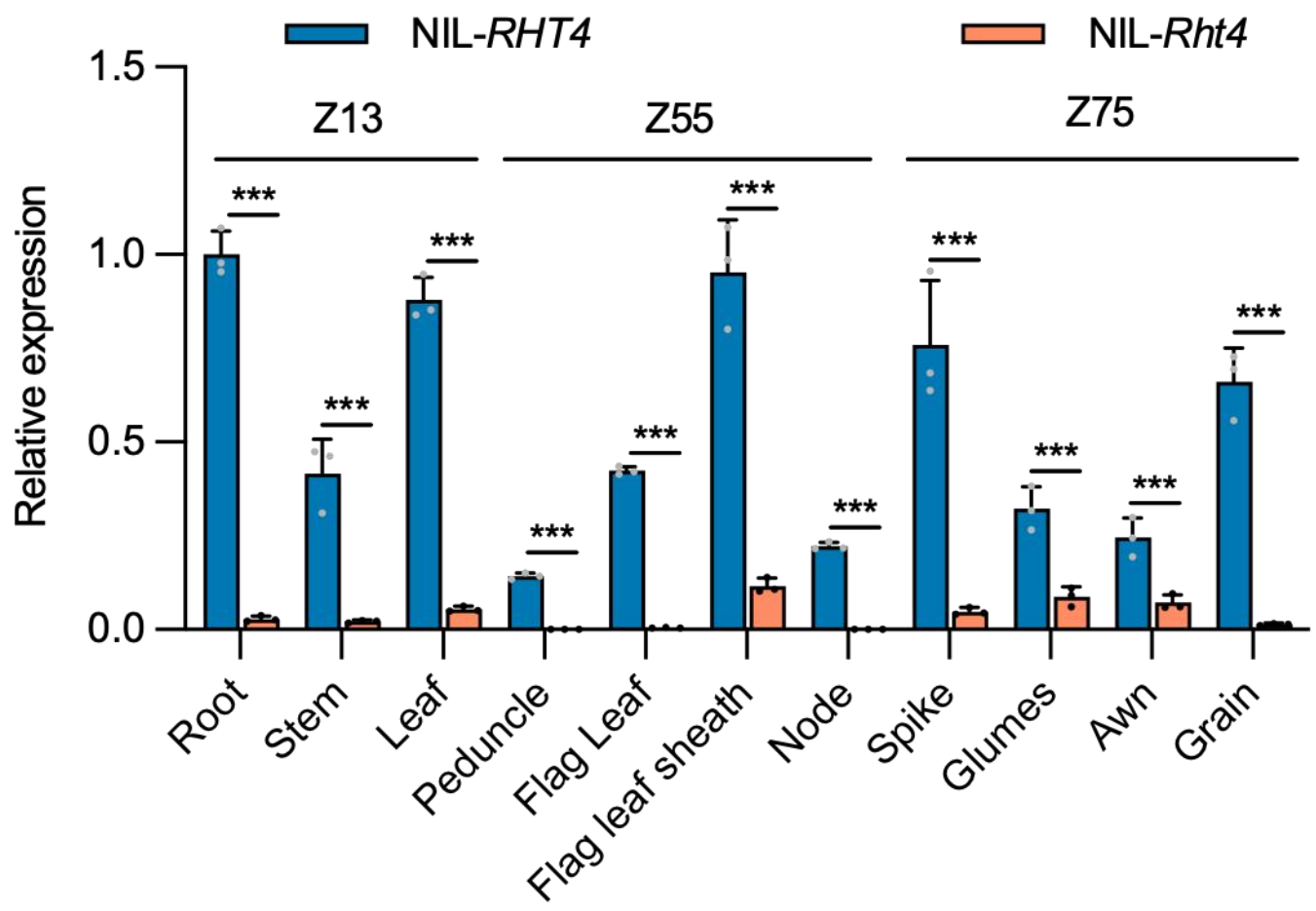

Figure S10 Expression patterns of *TaBSK3* in various tissues and organs in NIL-*RHT4* and NIL-*Rht4*.

Relative expression levels of *TaBSK3* in NIL-*RHT4* and NIL-*Rht4* at three-leaf stage (Z13), heading stage (Z55) and filling stage (Z75) were normalized to *TaActin*. Data are presented as means ± SD, $n = 3$. **P* < 0.05, ***P* < 0.01, ****P* < 0.001, Student's *t*-test.

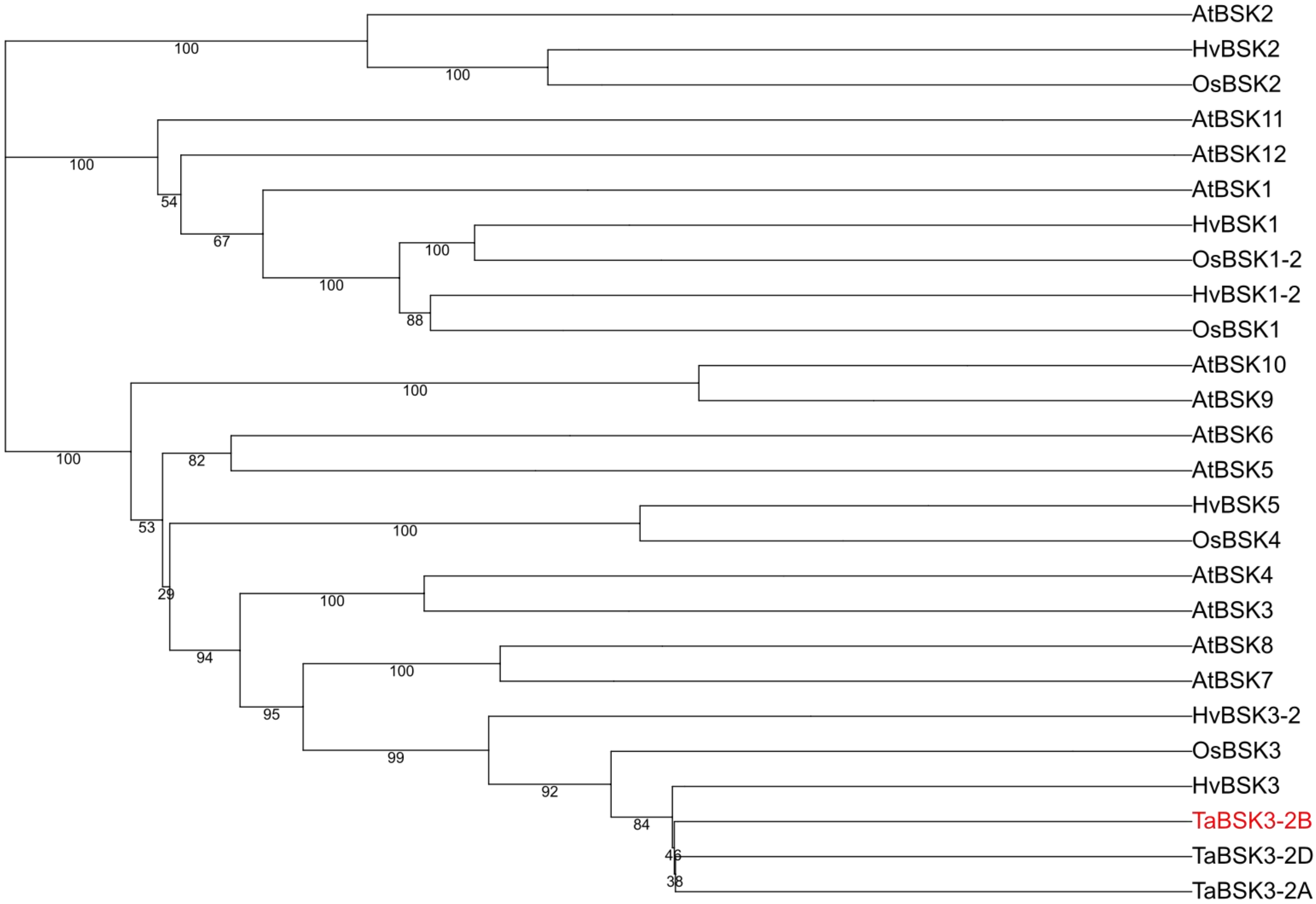

Figure S11 Phylogenetic analysis of BSK proteins across plant species.
Neighbor-joining tree was constructed using the full-length sequence of BSK proteins from *Triticum aestivum* (Ta), *Hordeum vulgare* (Hv), *Arabidopsis thaliana* (At) and *Oryza sativa* (Os) with MEGA 10. Poisson model was used to estimate amino acids substitution and bootstraps were based on 1,000 replications. Bootstrap values were shown over the branches.

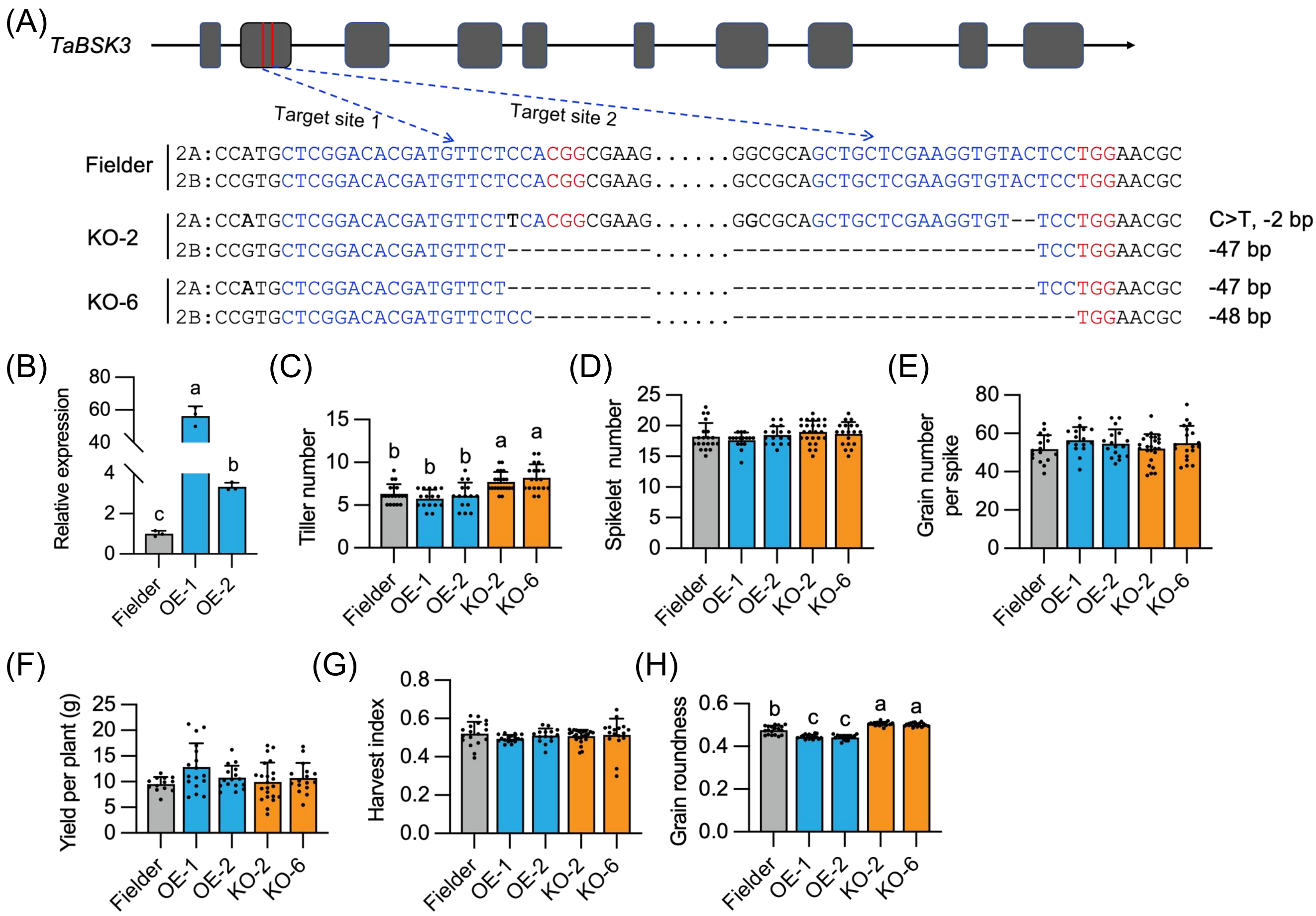

Figure S12 Molecular and phenotypic characterization of *TaBSK3-2B* overexpression (OE) lines and *TaBSK3* CRISPR-Cas9 engineered knockout (KO) mutants.
(A) Schematic illustration and sequence alignment of the two gRNA target sites of *TaBSK3*. CRISPR-Cas9 was used to generate *TaBSK3* knock out (KO) mutants. Blue bases represent the sgRNA target sequences and red bases indicate the protospacer adjacent motif (PAM) sequences. '–' indicates base deletions. The black boxes represent the coding regions of *TaBSK3*. (B) Relative expression of *TaBSK3* in Fielder, OE-1, OE-2, KO-2 and KO-6 lines. (C-H) Comparison of tiller number (C), spikelet number (D), grain number per spike (E), yield per plant (F), harvest index (G), and grain roundness (H) among Fielder, OE-1, OE-2, KO-2 and KO-6 lines. Data are presented as means ± SD. Different letters indicate significant differences at $P < 0.05$ (one-way ANOVA, Tukey's HSD test).

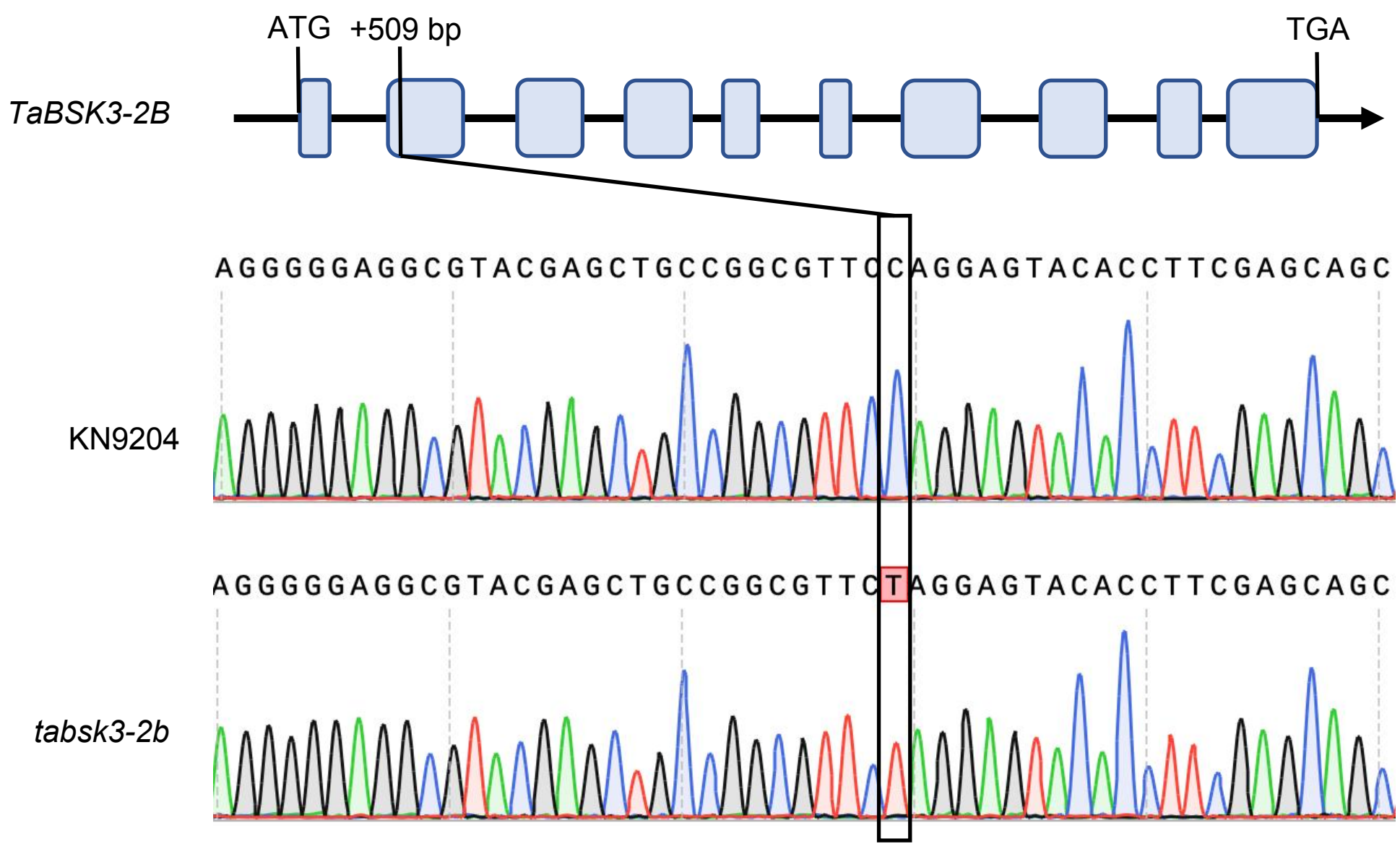

Figure S13 Sequence identification of *tabsk3-2b* mutant in KN9204 background.
Sanger sequencing chromatograms showing the mutation site of *TaBSK3-2B* in the *tabsk3-2b* mutant. The black box indicates the mutated nucleotide.

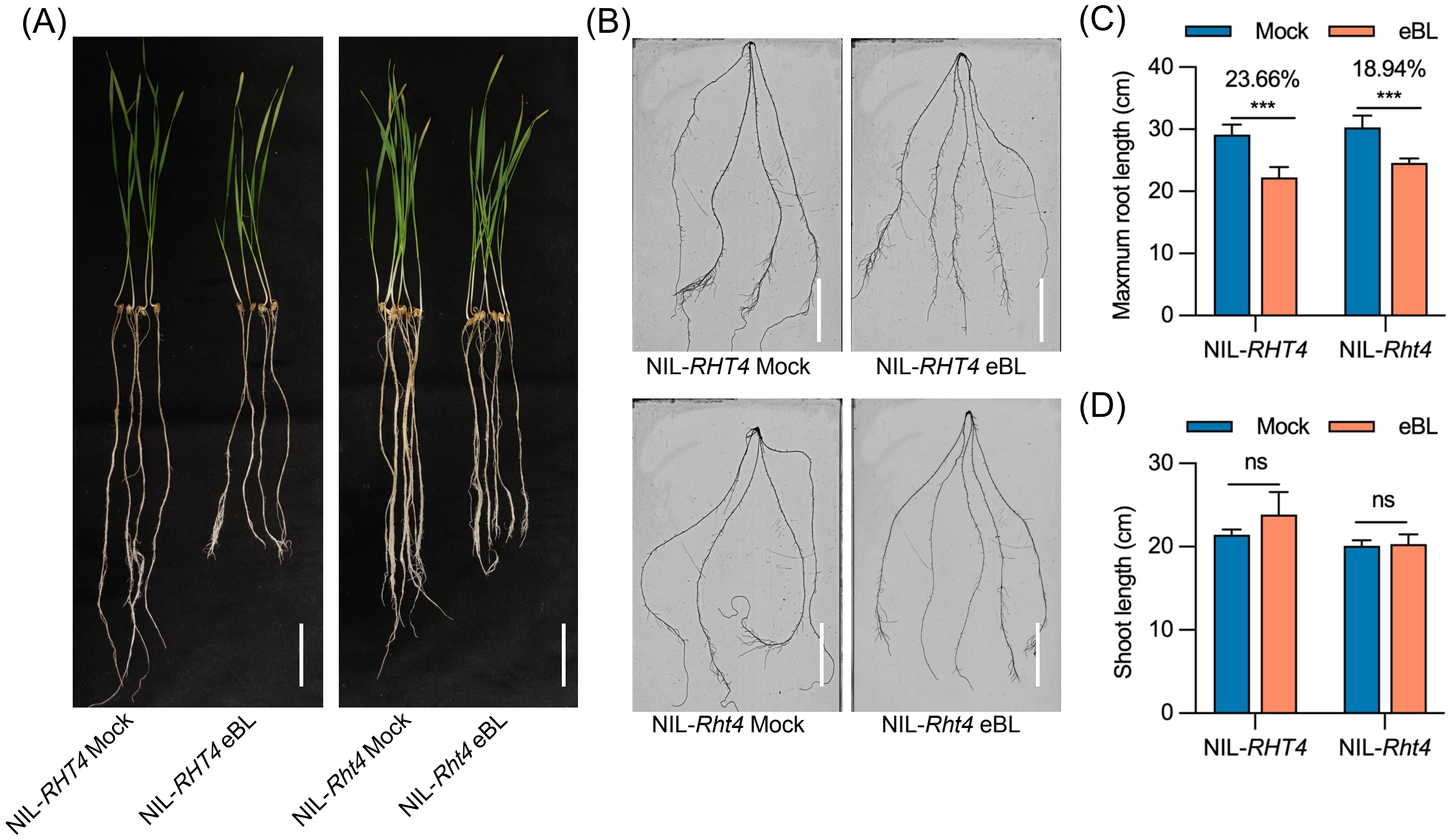

Figure S14 The growth responses of NIL-*RHT4* and NIL-*Rht4* seedlings upon eBL treatment. (A) Seedlings of NIL-*RHT4* and NIL-*Rht4* grown under eBL treatment. (B) Comparison of root length in response to exogenous eBL between NIL-*RHT4* and NIL-*Rht4* after a 10-day eBL treatment. Bars = 5 cm. (C-D) Statistical analysis of the maximum root length (C) and shoot length (D) after applying with eBL. Data are presented as means ± SD, $n = 10$. ***$P < 0.001$, Student's *t*-test.

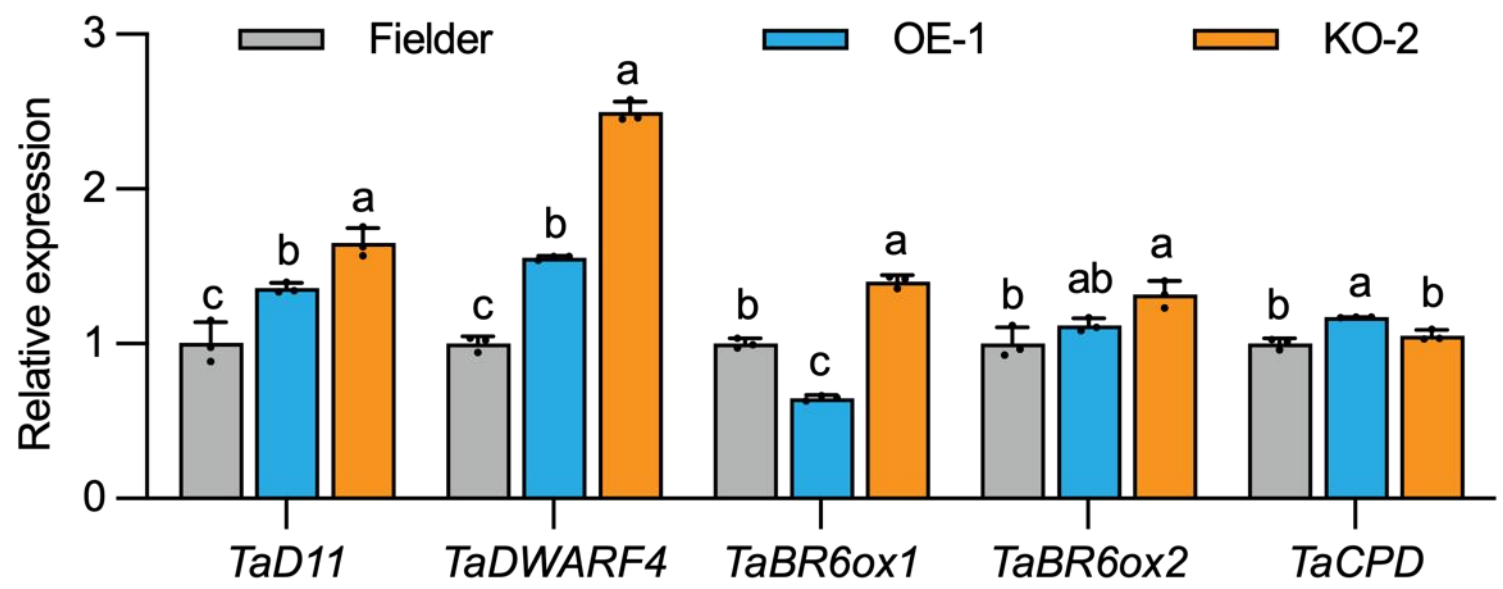

Figure S15 Expression patterns of BR biosynthesis-related genes in Fielder, *TaBSK3-2B* OE-1 and *TaBSK3* KO-2.

Relative expression levels of genes involved in BR biosynthesis pathway in peduncles of Fielder, *TaBSK3-2B* OE-1 and *TaBSK3* KO-2 at heading stage (Z55). Different letters indicate significant differences at $P < 0.05$, one-way ANOVA, Tukey's HSD test.

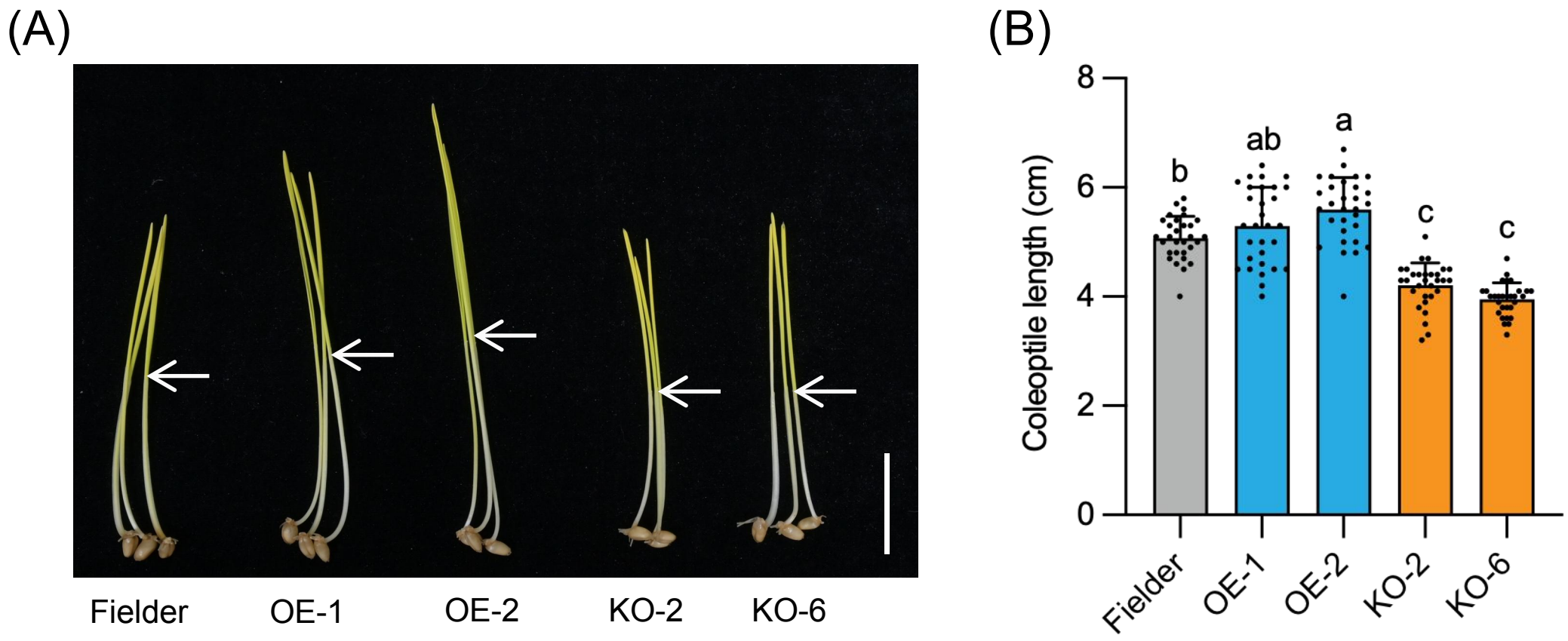

Figure S16 Coleoptile length of *TaBSK3-2B* overexpression (OE) and *TaBSK3* knockout (KO) lines.

(A) Comparison of coleoptile phenotypes of Fielder, *TaBSK3-2B* OE-1 and OE-2, *TaBSK3* KO-2 and KO-6 seedlings after germination and growth in darkness for 7 days. Arrows indicate the coleoptile tips. Bar = 3 cm. (B) Statistical analysis of the coleoptile length of Fielder, *TaBSK3-2B* OE-1 and OE-2, *TaBSK3* KO-2 and KO-6 lines. Different letters indicate significant differences at $P < 0.05$, one-way ANOVA, Tukey's HSD test.

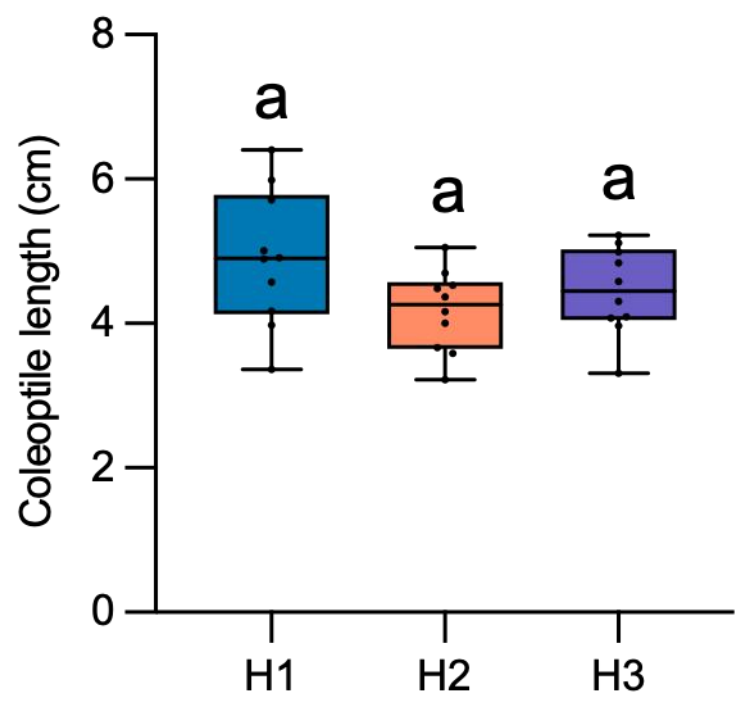

Figure S17 Coleoptile length of accessions carrying three haplotypes of *TaBSK3-2B*.

Comparison of the coleoptile phenotypes among accessions carrying three haplotypes of *TaBSK3-2B*. Each data point represents the BLUP value of coleoptile length about each accession. $n = 10$. Same letters indicate no significant differences at $P < 0.05$, one-way ANOVA, Tukey's HSD test.

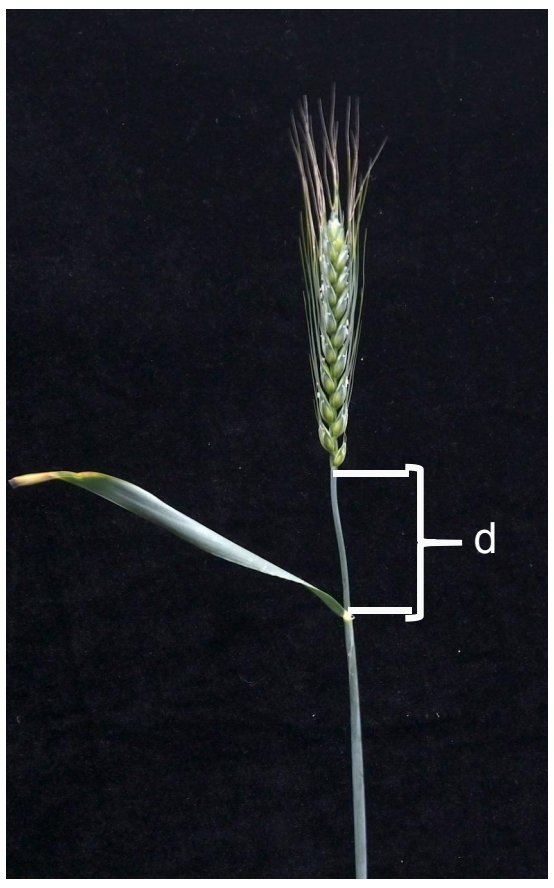

Figure S18 Definition of the spike-leaf distance in wheat.
Spike-leaf distance is defined as the vertical distance between the base of the spike and leaf stalk of the flag leaf on the main stem. As shown in the figure, the bracket labeled "d" indicates this interval.
