# Supplementary material for: Deletion of TaBSK3‐2B attenuates BR signaling to regulate the Rht4 semi‐dwarf phenotype and drought tolerance in wheat
Source: Plant J. 2026 Sep 17;127(5):e71118. doi: 10.1111/tpj.71118 (PMC13584235; doi:10.1111/tpj.71118)
Supplement: Supplementary file 2 [file TPJ-127-0-s002.pdf]

Table S1. Primers used for RT-qPCR assay.

Primer	Forward sequence (5'-3')	Reverse sequence (5'-3')
TaBSK3-qpcr	TCTCCTCTTGCTGACGCTTG	TGAGGTTAATGTGTCCTGCAT
TaGA20ox2	CCCTGGAAGGAGACCCTCT	GGGTGCTGGTGAAGTAGTCC
TaGA3ox2	AGGTCGCCGCCGTCGAGTCC	CAGTTGAGGTGCATTGTGGC
TaGA2ox3	GACCCCGGGGACTACTTCT	TTGACGAGCTTGAAGAACCC
TaEXB11-6A	GTATCATCAAGATCCAGTACAAGAGGG	ATTCGATCAGGACCTCCAGGTAGTA
TaEXB11-6D	GTCGCTCTACAGAGATGGCAAGG	TGCCGCTCATGTCGAAGTGG
TaEXP4	CCACAAGCATAAGCGCTGGA	GAGAAGGAAAGCGTCTAAGCG
TaBC1	ACAAACGCATCCATCCCTAC	TTCCAGTCGTCCATCCTCT
TaGT43A	TCTACGACACCCACTTCT	CCAGCCGACTACCTTCGA
TaWRKY71	CGTTACAGCGCTTGGTGTTT	CTCCGACTCAAGAACCGCAA
TaCYCB2	TCGGTCAGAGAGAGTGAGGG	GATGGATGCAGAGGAGGAGC
TaNRT1	ATCCACTGCTCCAGAGATCCA	GTGAGGTAGGTGACGAGGTTC
TaPLL	TCACTTGCACTTGCCTCCTC	CCCCATTTCGACCTACACGG
TaCOBRA5	GGATATCCAGTCATGGACGCC	TGGCCGTAGGCGGAGAT
TaPRR95	GGTGGTGGAGGCAAACTTGA	CAGAAGCTGCAGAAACGTGG
TaMYBL1	GAGGAGTTCCAGATCGACGACAGT	GCATGAACAGCGTGACCCAGA
TaP5CS1	TTGCTCACGGCATCTCCA	TGCCTTCAGGGTCATCCTAG
TaXERO1	GACTAACCGCGTCGACGAGTA	TCTCGTGGGCTGGAAATGC
TabZIP53	ATGGAGATGATTCCGCCTGCT	TGCCTTTTCATGCGAGATCTCT
TaGPX1	CGAGCTGAGCCAGCTGTAC	CCACCTTTGCTAGACTTCAGGA
TaGW2	ACTGAGGCTGTTAAGGGAGAAC	GACAGGGAGGTCCATTTCG
TaBR6ox1	AAGACGATGAGAGCGCGATT	ACAGGAGCATGTTGTTGCCT
TaBR6ox2	GACCCGATACAGATGGCAGG	TCTTTCCGTCGGTGCAGAAT
TaCPD	GCTCGTCTTCTTTCCCACCA	CCACACATTTTACAAGCAGCAC
TaD11	GCATACCCGTACGTGGAGTT	GCTAAGCTAGTCATCGGTGCTT
TaDWARF4	ACCAGGCGTTCGTGTACC	TCCCCTAGACGCTAGACTGAG
TaActin	GACCGTATGAGCAAGGAGAT	CAATCGCTGGACCTGACTC

Table S2. Primers used for map-based cloning.

Primers	Sequence (5'-3')	Type
W317-F	TGCTAGCAATGCTCCGGGTAAC	SSR
W317-R	TCACGAAACCTTTTCCTCCTCC	
I18-F	TGGAGAACTGCATATCTTAG	InDel
I18-R	GTCAGTTAGAATTCGTGTGA	
I35-F	ACGAGTAGGCGCTTGGCAGT	InDel
I35-R	AGGCACGGACCAGGTCAAAT	
K1-F1	TCTCATGGTACTGTACTTCTTCCAT	KASP
K1-F2	TCTCATGGTACTGTACTTCTTCCAG	
K1-R	TCCAGCCAAGGTTTGCCCTC	
K3-F1	ATGAACATGAAACTTGTCGAGGTTTAC	KASP
K3-F2	CATGAACATGAAACTTGTCGAGGTTTAA	
K3-R	CGAGAAAAGGTATACGTCATTATCCACAA	
K8-F1	CGTAGGCTGTAGTCCACATCG	KASP
K8-F2	CGTAGGCTGTAGTCCACATCA	
K8-R	AAAATAACAATCCATCGGCCTG	
K9-F1	AGTAATAAGCAACCTCTTCACTGAC	KASP
K9-F2	AGTAATAAGCAACCTCTTCACTGAT	
K9-R	TTCACGTCACCATGACCCATT	
K10-F1	TTTCTTTAGGCGAAGGAGAGATAA	KASP
K10-F2	TTTCTTTAGGCGAAGGAGAGATAG	
K10-R	CGATCAAAGTGCACCATGAC	
K11-F1	CCAGTGGCCTTAAGCTTCCG	KASP
K11-F2	CCAGTGGCCTTAAGCTTCCA	
K11-R	GCTCCGGGAATACTGAGGG	
K13-F1	TGAACGTGCTCCTGAATGAT	KASP
K13-F2	TGAACGTGCTCCTGAATGAC	
K13-R	ACCAAAATCCGAGACCTTTGC	
K16-F1	ACAGCACAAGTATGGAACTCA	KASP
K16-F2	ACAGCACAAGTATGGAACTCG	
K16-R	CACTGTCAACCTGCAACGTC	
K19-F1	AGCAGTCGTCCGTCCATA	KASP
K19-F2	AGCAGTCGTCCGTCCATC	

K19-R	GCAGATCGAGGGCCTCCC	
K26-F1	ATTGCTTGGGGCGCCTCA	
K26-F2	TGACTCTGTCATCTCCCTTCTC	KASP
K26-R	GGTGTCTGATCCGTGGCG	
K31-F1	AGGCCATAGCAACACCTTCG	
K31-F2	AGGCCATAGCAACACCTTCA	KASP
K31-R	ACGGTGACACTAACTAGCGC	
K33-F1	AATGTTGGCCTGCCCGGT	
K33-F2	AATGTTGGCCTGCCCGGC	KASP
K33-R	GGGTCACATCCGGCTAAACT	
K34-F1	CAATCGCAGCCACAACGTA	
K34-F2	CAATCGCAGCCACAACGTT	KASP
K34-R	CATAATGATGACCGTGCGGC	
K46-F1	TGGGTGCTTTGTAGTTGTTGA	
K46-F2	TGGGTGCTTTGTAGTTGTTGG	KASP
K46-R	GATACAAGGGTGTCGGGCAC	
K47-F1	CCACCTGAAGCACTCTGCC	
K47-F2	CCACCTGAAGCACTCTGCT	KASP
K47-R	CAGTGGCCTCTCGCTGATC	
K49-F1	ACCACATAACGACAACCCTCA	
K49-F2	ACCACATAACGACAACCCTCG	KASP
K49-R	CGGCTTTAGCACGACTGACT	

Table S3. Primers used for plasmid construction and genotyping.

Primers	Sequence (5'-3')	Description
TaBSK3-2B-cds-F	ATGGGCGCGCGCGTGTCC	Gene cloning
TaBSK3-2B-cds-R	TCAGCGTCCGCTGCTGCTACTTGTC	
TaBSK3-2B-OE-F	gaaaaagagggggattaactagtATGGGCGCGCGCGTGTCC	Construction of overexpression and CRISPR/Cas9 vector
TaBSK3-2B-OE-R	accgctaccaccgctaccgagctcGCGTCCGCTGCTGCTACTTGTC	
TaBSK3-sgRNA1	GGAGTACACCTTCGAGCAGC	
TaBSK3-sgRNA2	TGGAGAACATCGTGTCCGAG	
TaBSK3-2B-1302-F	gggactcttgaccatggtaATGGGCGCGCGCGTGTCC	Subcellular localization
TaBSK3-2B-1302-R	ctcaccatcctaggactagtGCGTCCGCTGCTGCTACTTGTC	
TaBSK3-2B-16318h-F	cttgcatgcctgcaggtcgacATGGGCGCGCGCGTGTCC	
TaBSK3-2B-16318h-R	gcccttgctcaccatggatccGCGTCCGCTGCTGCTACTTGTC	
TaBSK3-2B-nYFP-F	atcgaggacgccggcggatccATGGGCGCGCGCGTGTCC	BiFC assays
TaBSK3-2B-nYFP-R	acgaaagctctgcaggtcgacTCAGCGTCCGCTGCTGCTACTTGTC	
TaBSU1-1A-cYFP-F	attacaggtacccggggatccATGGGGACGGCGGGGAA	
TaBSU1-1A-cYFP-R	cacgctgccaccgccgtcgacTATGTAAGCAAGAGAGTTCC	
TaBSK3-2B-Cluc-F	ggtggaagtggtggatccATGGGCGCGCGCGTGTCC	LCI assays
TaBSK3-2B-Cluc-R	tccagatccaccgtcgacGCGTCCGCTGCTGCTACTTGTC	
TaBSU1-1A-Nluc-F	ggtggaagtggtggatccATGGGGACGGCGGGGAA	
TaBSU1-1A-Nluc-R	tccagatccaccgtcgacTATGTAAGCAAGAGAGTTCC	
Hitom-seq-F	ggagtgagtacggtgtgcCCTTGTAGACGACGTTGG	Identification of gene-editing mutation sites
Hitom-seq-R	gagttggatgctggatggGTTGAGCCCAAGGGTTAT	

Table S4. Information of the three deleted genes within the mapping interval.

Gene ID	Description	Rice ortholog	Arabidopsis ortholog	Reference
TraesCS2B03G1511100	Pentatricopeptide-repeat region of PRORP	*OsWSL5* (*Os04g0684500*)	*AT5G04810*	Liu et al., 2018
TraesCS2B03G1511400	Amidase	*Os04g0118100*, *Os04g0117900*	*AT1G08980*	
TraesCS2B03G1512100	Serine/threonine-protein kinase	*OsBSK3* (*Os04g0684200*)	*AT1G01740*	Zhang et al., 2016 Tian, Xia, et al., 2022

Table S5. Information of 271 spring wheat accessions.

Source	H1	H2	H3
China	130	86	9
Canada	1	1	0
Kazakhstan	7	0	10
Australia	18	8	1
Landrace	47	2	0
Cultivar	86	84	18
Advanced breeding lines	23	9	2

Table S6. Sequence variations of the three major haplotypes of *TaBSK3-2B*.

	Position	H1	H2	H3
P1	796055741	-	-	CCCT
P2	796055696	CCCTG	-	CCCTG
P3	796055573	CTGCTCCTGCCACGCTGT ACCGTGCACGCCAGCCTG TACGCCCGTACGCACGTA GTACGTGC	CTGCTCCTGCCACGCTGT ACCGTGCACGCCAGCCTG TACGCCCGTACGCACGTA GTACGTGC	-
P4	796055535	T	T	A
P5	796055528	C	C	T
P6	796055521	G	G	T
P7	796055469	-	-	GA
P8	796054417	TAC	TAC	-
P9	796054001	C	A	C
Num. of accession		156	95	20
Cultivar		86	84	18
Landrace		47	2	0
Advanced breeding lines		23	9	2

Table S7. Accession numbers of the genes in this study.

Gene symbol	IWGSC RefSeq v2.1		
TaBSK3-2B	*TraesCS2B03G1512100*		
TaBSU1-1A	*TraesCS1A03G0213700*		
TaCYCB2	*TraesCS2A03G0973200*	*TraesCS2B03G1077200*	*TraesCS2D03G0912100*
TaEXB11-6A	*TraesCS6A03G0675300*		
TaEXB11-6D	*TraesCS6D03G0566000*		
TaNPF6-S3	*TraesCS4D03G0829000*		
TaEXP4	*TraesCS1A03G0748300*	*TraesCS1B03G0853100*	*TraesCS1D03G0716600*
TaCOBRA5	*TraesCS5A03G0240400*	*TraesCS5B03G0257900*	*TraesCS5D03G0253700*
TaGA2ox3	*TraesCS3A03G0732000*	*TraesCS3B03G0838600*	*TraesCS3D03G0675100*
TaGA3ox2	*TraesCS3A03G0268400*	*TraesCS3B03G0336700*	*TraesCS3D03G0261200*
TaGA20ox2	*TraesCS3A03G0950800*	*TraesCS3B03G1087000*	*TraesCS3D03G0884400*
TaBC1	*TraesCS5A03G0570800*	*TraesCS5B03G0582100*	*TraesCS5D03G0536900*
TaGT43A	*TraesCS1A03G0155800*	*TraesCS1B03G0205900*	*TraesCS1D03G0143400*
TaWRKY71	*TraesCS6A03G0350400*	*TraesCS6B03G0446800*	*TraesCS6D03G0304200*
TaPRR95	*TraesCS5A03G0778000*	*TraesCS5B03G0809900*	*TraesCS5D03G0735000*
TaMYBL1	*TraesCS6A03G0618800*	*TraesCS6D03G0523500*	
TaP5CS1	*TraesCS3A03G0864700*	*TraesCS3B03G0987700*	*TraesCS3D03G0793300*
TaXERO1	*TraesCS6A03G0901400*	*TraesCS6D03G0772700*	
TabZIP53	*TraesCS4A03G0247900*	*TraesCS4B03G0521300*	*TraesCS4D03G0460100*
TaGW2	*TraesCS6A03G0480200*	*TraesCS6B03G0578500*	*TraesCS6D03G0404800*
TaGPX1	*TraesCS2B03G1093200*	*TraesCS2D03G0923200*	
TaActin	*TraesCS5A03G0347200*	*TraesCS5B03G0340400*	*TraesCS5D03G0333200*
